# Vertical farming as a land sparing strategy: GHG implications for UK agricultural landscapes

**DOI:** 10.1016/j.clfs.2026.100037

**Published:** 2026-06

**Authors:** Michael Gargaro, Kaiwen Li, Richard J. Murphy, Astley Hastings, Zoe M. Harris

**Affiliations:** a Centre for Environment and Sustainability, University of Surrey, Guildford, England, GU2 7XH, UK; b University of Aberdeen, King's College, Aberdeen, Scotland, AB24 3FX, UK

**Keywords:** Life cycle assessment, Field farming, Vertical farming, Land sparing, Systems modelling, Lettuce, GHG emissions

## Abstract

Growing pressure on UK land, driven by demands for food production, renewable energy, biodiversity recovery and climate mitigation, is intensifying competition for limited land. Vertical farming (VF) offers a land-sparing strategy: by relocating crop production indoors and achieving extremely high yields per unit area, VF can free agricultural land for other uses. However, VF has high greenhouse gas (GHG) emissions compared to traditional field farming due to intensive electricity demand, so its environmental value depends on whether land-sparing outcomes can offset production-stage impacts.

This study provides the first UK-wide, full-system assessment of lettuce production, comparing business-as-usual field farming with national-scale VF and alternative uses of spared land. A primary-data life cycle assessment (LCA) quantifies the impacts of VF; a separate primary-data LCA of field farming incorporates DNDC-modelled national soil emissions; and the Land Use Net-Zero Advisor (LUNA) evaluates the GHG, land-carbon and energy implications of repurposing spared land for solar, wind, afforestation, agroforestry and bioenergy.

VF reduces land demand by 93% but has higher GHG emissions per-kg than field systems. System-level outcomes depend on how spared land is used. Solar energy provides the strongest mitigation, fully offsetting VF's operational emissions and reducing total system impacts below the field baseline. Forestry and agroforestry generate positive land-carbon outcomes but more modest GHG reductions. Across all scenarios, peat soils dominate land-carbon losses, underscoring the need to avoid land-use change on peat and prioritise restoration.

Overall, VF's environmental value arises from the land-use transitions it enables. When incorporated into multifunctional land-use planning, VF can support domestic food production while facilitating renewable-energy deployment and ecosystem restoration within a highly constrained UK land system.

## Highlights

• Full system LCA compares field lettuce with vertical farming and use of spared land.
• Vertical farming enables major land sparing through very high yields per unit area.
• Vertical farming emissions exceed field farming due to high electricity use.
• Solar on spared land reduces total system emissions to below the field baseline.
• Avoiding peat soils is critical to reducing land use emissions.

## 1 Introduction

Limiting global temperature rise to below 2 °C depends on maintaining atmospheric carbon dioxide (CO_2_) eq. concentrations under 450 ppm (Reilly et al., 2012). Achieving this will require multiple, concurrent strategies for climate mitigation, many of which involve land use change (LUC). However, land-based mitigation strategies must contend with rising demands for food and forest products, alongside numerous other societal land uses, driven by rapid population growth (Reilly et al., 2012; IPCC, 2019; Searchinger et al., 2023). The World Resources Institute projects that an additional 600 million hectares of agricultural land will be needed by 2050 to meet the increases in demand for crops (56%), meat and milk (70%), and wood (54%) (Searchinger et al., 2023). These trends highlight the urgent need for innovative solutions that balance feeding a growing population with preserving and enabling the natural world's ability to deliver essential ecosystem services.

Like many nations globally, the UK faces increasing pressure to balance agricultural production with climate and environmental mitigation commitments, environmental restoration goals and the demands of a growing population. With the introduction of the Land Use Framework into UK policy, there is renewed urgency to ensure that land is used to its fullest potential (DEFRA, 2025b). Multiple competing demands now converge on the same finite land resource; all aimed at meeting a wide range of outcomes (DEFRA, 2025b; CCC, 2020). For example, Environment Act targets and the UK's Net Zero by 2050 commitment imply at least 1.6 million hectares of LUC by 2050 (DEFRA, 2025b). These changes include goals such as restoring 280,000 hectares of peatland and increasing tree and woodland cover to 16.5% from 14% of total land area by 2050, among many others, illustrating the scale and diversity of competing land demands (DEFRA, 2025c).

Crucially, the UK aims to achieve these transformations without reducing domestic food production (DEFRA, 2025b), an objective recognised by the UK Climate Change Committee as a key constraint on land-based mitigation pathways (CCC, 2020). In 2023, the utilised agricultural area accounted for 70% of UK land, producing 58% of the food consumed by value (Cousins, 2021; DEFRA, 2024), however, the UK remains ‘food secure’ by sourcing from a diverse range of stable regions alongside domestic supply. Nonetheless, both climate change and global market volatility have already demonstrated their capacity to disrupt food production and prices. The 2008 food price spike, driven by extreme weather and trade instability, saw wheat prices rise by 130%, while the 2018 UK heatwave caused widespread crop spoilage, leading to a 22% increase in lettuce price due to reliance on imports from the US and Spain (Butler, 2018; Cousins, 2021; Gargaro et al., 2025). With global temperatures projected to rise further and extreme climatic events becoming more frequent and severe (IPCC, 2019), safeguarding food production while addressing vulnerabilities in the food system is critical. This dual challenge of meeting environmental targets without compromising food security underscores the need for integrated, innovative land-use strategies.

Vertical farming (VF), a form of controlled environment agriculture, may provide a solution to these challenges. VF is a technology-driven approach to crop production that optimises key production inputs and resource use, including lighting, heating, water, nutrient delivery, energy, and space. These systems create optimised growth conditions while shielding crops from external stressors (Gargaro et al., 2023, 2025; Kozai and Niu, 2015). Currently, VF systems primarily cultivate leafy greens and salad crops, leveraging their compact size (typically under 30 cm) and higher market value (Birkby, 2016; Kozai and Niu, 2016). This study focuses on UK lettuce production, a crop widely grown in VF systems yet heavily imported to the UK for much of the year (Jordan, 2020).

Recent studies comparing VF with conventional field farming reveal that VF has a higher climate change impact (Blom et al., 2022; Casey et al., 2022; Gargaro et al., 2025). The primary driver of VF's environmental impact is its high electricity demand, which can account for 86–91% of its climate change footprint when powered by grid electricity (Gargaro et al., 2024, 2025; Sandison et al., 2023). However, growing competition for land, driven by the need to simultaneously maintain food production, meet climate targets and deliver environmental restoration, has intensified interest in production systems that reduce reliance on conventional agricultural land (IPCC, 2022; Kalantari et al., 2018; Searchinger et al., 2023). In this context, VF offers advantages when considered within the broader context of land-use pressures. By utilising vertical space, VF can dramatically increase yields per unit of land area. Research indicates VF can achieve lettuce yields of 95–101 kg m^−2^ yr^−1^, approximately 25 times higher than traditional field farming (Blom et al., 2022; Gargaro et al., 2025; Touliatos et al., 2016). This provides an opportunity to alleviate the pressure of competing land demands by relocating cultivation indoors, enabling the sparing of land by concentrating production on a smaller land footprint. Thus, the land-sparing potential of VF could strengthen food security while freeing land for other environmental and climate objectives.

The climate benefits of land sparing arise from the alternative uses enabled on freed land. IPCC mitigation pathways consistently identify woodland expansion, peatland restoration, bioenergy cropping and renewable energy deployment as key land-based strategies for meeting 1.5–2 °C targets (IPCC, 2019; IPCC, 2022). Woodlands and peatlands represent some of the most important terrestrial carbon stores, providing long-term sequestration through biomass, while perennial bioenergy crops can supply low-carbon energy and, when combined with carbon capture and storage, contribute to net carbon removal (IPCC, 2019; IPCC, 2022). Renewable energy infrastructure further supports decarbonisation by displacing fossil-fuel electricity generation (IPCC, 2022). These established mitigation pathways provide a clear basis for evaluating the potential climate benefits of VF-enabled land sparing.

Although greenhouse lettuce production is an important component of UK horticulture also, this study focuses on a comparison between VF and open-field systems. Spatially explicit data on the location and extent of protected environments are not available at a national scale, limiting their inclusion in land-sparing analysis. Moreover, greenhouses typically involve permanent infrastructure (often with hardstanding), meaning their displacement would be unlikely to release land for alternative environmental or climate-mitigation uses. Open-field production therefore provides the most appropriate baseline for assessing the land-sparing potential of VF.

### 1.1 Aims

The research quantifies the environmental impacts (specifically climate change impacts) of adopting VF at a national scale in the UK, assessing the implications of replacing traditional field-based lettuce production with VF to meet domestic demand. Building on our previous work comparing the environmental performance of VF and conventional field farming in the UK and Spain (Gargaro et al., 2025), the present study expands its scope to include the land-sparing potential of VF and the environmental trade-offs arising from alternative uses of spared land. By examining whether VF can reduce land pressure for lettuce production in the UK context, and how any land spared could be used to achieve improved environmental outcomes, the analysis provides the first structured and data-rich assessment in the UK context that can inform broader debates around the use of VF, food production, and the efficient use of land. Although centred on a UK case study, the analytical approach, methodology, underlying questions, and insights are intended to be transferrable to other crops and regions.

## 2 Methodology

### 2.1 Methodological framework

**Fig. 1:**
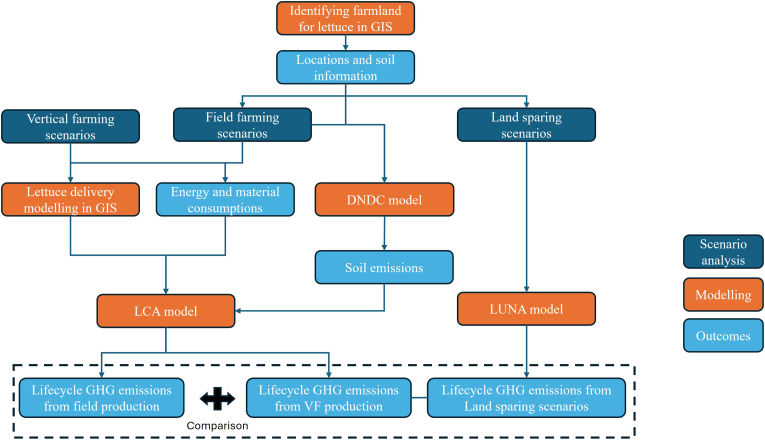
Overview of the methodological workflow performed. DNDC (Denitrification-Decomposition model), LCA (life cycle assessment), LUNA (Land Use Net Zero Advisor Tool).

A spatially explicit framework was developed to assess trade-offs in greenhouse gas (GHG) emissions across alternative lettuce production and land-use pathways in a UK national-scale context. The study identified the land most likely to be used for lettuce cultivation in the UK, followed by scenario development for field production, VF, and land sparing. Life cycle assessment (LCA) serves as the core method in this framework to compare the environmental impacts of field-based lettuce production and VF, incorporating soil emissions of field production from the Denitrification-Decomposition (DNDC) model into the LCA (DNDC, 2018). The life cycle environmental consequences of alternative uses of VF-spared land are modelled using the LUNA (Land Use Net-Zero Advisor) tool (Hastings et al., in preparation). Modelling results from the LCA of VF and LUNA are then combined, and compared with the LCA of field farming production (Fig. 1).

### 2.2 Land use context and scenario definition

Approximately 4,000 ha of UK land were used for lettuce cultivation in 2021, producing 88,000 tonnes (DEFRA, 2025a). Including protected environment cultivation (e.g. glasshouses and polytunnels), total UK production reaches 103,100 tonnes, supplemented by 201,700 tonnes imported and 4,000 tonnes exported, resulting in a net 300,800 tonnes of lettuce procured annually for domestic consumption (see Supplementary Section A for further details).

As no explicit maps of UK lettuce cultivation were available to the authors, the most likely land that could be used for lettuce production were estimated by overlaying field parcels of “other vegetables” from the UKCEH crop map with Agricultural Land Classification (ALC) grade 1 land, the land most commonly used for lettuce production in the UK (Natural England, 2025; UKCEH, 2025; WelshGov, 2021). Due to the rotational nature of UK cropping systems and the absence of spatially explicit lettuce location data, field parcels were randomly allocated within “other vegetables” and grade 1 land as representative lettuce fields, consistent with the widespread use of rotational cropping systems in UK agriculture (Upcott et al., 2023).

Field farming scenarios

Two UK field farming scenarios were examined (mineral soils and peat/organic soils). The data for these scenarios is taken from Gargaro et al. (2025), which provided reference life cycle inventory data for cultivation inputs, transportation of inputs, and waste management, and has been updated in this analysis with new soil and transportation of produce emissions profiles, derived from where in the UK lettuces originate. Soil emissions were calculated for each field parcel using the DNDC model (DNDC, 2018), and transportation impacts were estimated using the UK road network to determine shortest routes from farms to distribution centres, and onward to supermarkets, with average distances applied as model assumptions (see Supplementary Sections B and C).

In addition to domestically produced lettuce, the impact of Spanish grown lettuce for export to the UK was evaluated; results for the impact of imported lettuce are taken from Gargaro et al. (2025). Further details on imported lettuce production are available in Supplementary Section D.

Vertical farming scenarios

Two VF scenarios were examined to understand the comparative impacts between current business-as-usual field farming and VF systems, to cultivate lettuce for the UK market. Both VF scenarios are powered using 100% renewable energy, modelled as low-emission electricity generation in the LCA. However, LUNA differs in that it includes the full lifecycle emissions for energy generation, including infrastructure, and avoided emissions are benchmarked against the projected average UK grid electricity carbon intensity for 2025 (HM Government, 2023).

The two scenarios are as follows.

• Hyperlocal: VF facilities located within or near population centres and urban areas.
• Distribution-centre: VF facilities positioned adjacent to existing or newly designated UK distribution hubs.

In the hyperlocal scenario, VF facilities were located to minimise lettuce produce transportation distances by siting production close to population centres. Using 2021 census data on population size and local authority population density data (Carnell and Tomlinson, 2025; ONS, 2025), facilities were sized to supply up to 50,000 people per local authority, resulting in 1515 facilities nationwide.

In the distribution-centre scenario, VF facilities were located to reflect business-as-usual retail distribution and logistics patterns. Facilities were concentrated within major logistics hubs, primarily the UK “golden logistics triangle^1^”, supplemented by depots in large cities and remote regions (ONS, 2022). Based on existing UK supermarket distribution networks, 70 facilities were sited, each supplying up to one million people. Additional details of facility siting are provided in Supplementary Section E.

Transportation impacts for both scenarios were estimated using the UK road network to calculate shortest routes between VF facilities and supermarkets, with the national average distance lettuce must be transported from VF facilities to supermarkets applied as a model assumption. The hyperlocal scenario assumed lettuce produce delivery by electric bike courier direct from vertical farms to supermarkets, while the distribution-centre scenario assumed lettuce produce delivery by 16–25 tonne lorries, also direct from vertical farms (which are co-located with distribution centres) to supermarkets (see Supplementary Section B for more details). Life cycle transportation impacts were separated into two components and calculated: (i) all transport impacts excluding lettuce produce delivery to supermarkets, including procurement of inputs and waste transport, and (ii) impacts associated with delivery of lettuce produce to supermarkets. Life cycle inventory data were drawn from the authors’ previous VF studies (Gargaro et al., 2024, 2025), with methodological consistency maintained but adjustments made to reflect national-scale distribution.

Land sparing scenarios

Multiple land sparing scenarios were evaluated in this study, using spatial GIS data layers of GHG emissions, energy and products produced, and soil and biomass carbon changes caused by changing land use for renewable energy. These layers were developed for use in the Ecosystem Land Use Model (ELUM) model (Pogson et al., 2016) and were updated and incorporated into the new LUNA model employed in this paper, developed by the University of Aberdeen and Forest Research for DEFRA in the NCF ETPP-33 C10 project (Hastings et al., in preparation). In this model the underlying data is derived from the MiscanFor model for Miscanthus and Willow (Hastings et al., 2009; Shepherd et al., 2023), for forestry using Ecological Site Classification (ESC) (Pyatt et al., 2001) and CARBINE (Jenkins et al., 2012; Matthews et al., 2015), wind energy from WindFor (Von Hellfeld et al., in press) and solar from Carvalho et al. (2025). The driving data for these models was the climate from the UKCP18 CHESS-SCAPE RCP 6.0 Variant 1 climate data (Robinson et al., 2023) and soil data from the Harmonized World Soil Database (FAO, 2008).

The land sparing scenarios are introduced in Table 1. The system boundaries used to assess these scenarios are explained in Section 2.3.

**Table 1:** The land sparing scenarios evaluated in this study.

Land sparing scenario	Sub-scenario	Scenario description
***Miscanthus***	• Unabated energy generation • BECCS	The spared land is allocated to *Miscanthus* cultivation, with the harvested biomass used for electricity generation in both unabated power plants and BECCS facilities.
**Short Rotation Coppice (SRC) willow**	• Unabated energy generation • BECCS	The spared land is allocated to SRC willow cultivation, with the harvested biomass used for electricity generation in both unabated power plants and BECCS facilities.
**Solar energy**	n/a	The spared land is allocated to ground mounted solar arrays.
**Onshore wind energy**	• 2 MW turbines • 4 MW turbines • 6 MW turbines	The spared land is allocated to 2 MW, 4 MW, and 6 MW wind farms.
**Short Rotation Forestry (SRF)**	• Unabated energy generation • BECCS	The spared land is allocated to SRF, with the harvested biomass used for electricity generation in both unabated power plants and BECCS facilities.
**Agroforestry**	• Unabated energy generation • BECCS	The spared land is allocated to agroforestry systems, with the harvested woody biomass used for electricity generation in both unabated power plants and BECCS facilities.
**Broadleaved forest-Continuous Cover Forestry (CCF)**	• Unabated energy generation • BECCS	The spared land is allocated to broadleaved forests, managed under CCF, with the harvested biomass used for electricity generation (and wood product streams) in both unabated power plants and BECCS facilities.
**Broadleaved forest-Standard Thin, Standard Fell (STSF)**	• Unabated energy generation • BECCS	The spared land is allocated to broadleaved forests, managed under STSF, with the harvested biomass used for electricity generation (and wood product streams) in both unabated power plants and BECCS facilities.
**Conifer forest CCF**	• Unabated energy generation • BECCS	The spared land is allocated to conifer forests, managed under CCF, with the harvested biomass used for electricity generation (and wood product streams) in both unabated power plants and BECCS facilities.
**Conifer forest STSF**	• Unabated energy generation • BECCS	The spared land is allocated to conifer forests, managed under STSF, with the harvested biomass used for electricity generation (and wood product streams) in both unabated power plants and BECCS facilities.

Each scenario was run in LUNA, and outputs were exported to GIS software for analysis.

A scenario was also run to examine the impact of LUCs specifically on mineral soils. All land-use scenarios initially appeared as net land carbon emitters due to the inclusion of peat soils, which possess very high baseline soil organic carbon (SOC) stocks and are highly sensitive to disturbance. To isolate mineral soil responses, peat soils were removed from the analysis. We additionally evaluated the climate change impact of restoring the peat soil area originally used for lettuce, to provide a reference case for peatland recovery relative to land-use conversion.

### 2.3 LCA methodology

Environmental LCA is a systems-analysis tool to display the potential environmental impact profile of products and services across a range of environmental impact categories. The LCA methodology developed is based on ISO standards 14040 and 14044 (ISO, 2006a, 2006b), to compare the life cycle (cradle-to-supermarket) environmental impact of field farming with VF and land sparing scenarios.

Goal and scope

The goal of the LCA research was to model the environmental impacts (climate change impacts) of current field-based lettuce production in the UK (and imported) compared with a scenario where VF systems supply all domestic lettuce demand, and spared land is repurposed for environmental objectives. The aim is to determine whether alternative uses of spared land due to VF adoption at a national scale result in improved environmental outcomes compared with conventional field lettuce production and procurement for the UK.

**Fig. 2:**
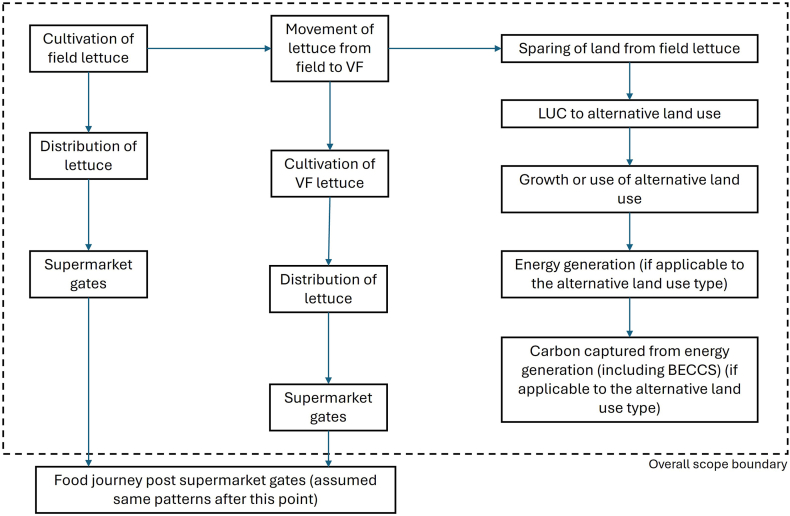
Overall scope and system boundaries of study. Detailed system boundaries for field farming, VF and land sparing scenarios are given in Supplementary Section F.

The scope encompasses all operational stages of both VF and field farming systems; from input delivery through cultivation and harvesting to distribution of crops to UK supermarkets. Additionally, the analysis incorporates LUC impacts resulting from replacing field farming with alternative land uses, and evaluates the associated impacts through 2050. See Fig. 2 for an overview of the overall scope of this study.

Functional unit

The functional unit for all scenarios is “The production of 1 kg of marketable lettuce delivered to supermarkets in the UK”. The functional unit represents lettuce produced and transported to supermarkets, where 90% of UK lettuce purchases occur (Laughton, 2023). “Marketable lettuce” refers to lettuce that meets UK marketing standards as defined under Commission Implementing Regulation (EU) No 543/2011 (EU, 2011).

This functional unit incorporates land-sparing impacts by assigning the environmental impacts of alternative land uses to each kilogram of lettuce. This ensures that differences between land sparing scenarios reflect both production impacts and the implications of how spared land is used. Results are applied consistently across scenarios and are also reported at the national scale, expressed as total marketed lettuce or total land area spared. While the functional unit remains 1 kg of marketable lettuce for all LCA-based comparisons, scenario-level analyses, particularly those relating to land sparing and land-carbon change, also report results in absolute terms (e.g., total GHG emissions or total carbon change) to reflect whole-system, national-scale outcomes. All LCAs however are performed per kg of marketable lettuce, but aggregated to national totals by scaling to UK demand over 25 years.

System boundaries

This cradle-to-supermarket assessment covers the full life cycle of current field farming and VF, including the production and delivery of system inputs, cultivation (including soil management activities), waste management, packaging and cleaning operations, delivery to supermarkets, and the use of spared UK land, accounting for associated LUC impacts.

Environmental burdens related to supermarket and consumer use and disposal are assumed equivalent to existing food operations and are excluded, as are embodied emissions from capital goods (buildings, machinery etc) and human labour and employee transport. System boundaries therefore commence from input production and end with delivery to retailers. Detailed system boundaries for field farming, VF, and spared land are provided in Supplementary Section F.

For consistency with previous VF LCAs performed, embodied emissions from capital goods (e.g. buildings and production infrastructure) are excluded from the VF and field farming systems, reflecting the current lack of robust primary data for VF facility construction. In contrast, land-use pathways modelled in LUNA include the full life-cycle impacts of new infrastructure associated with alternative land-sparing options, such as solar installations and wind turbines.

Soil emissions modelling (DNDC)

Soil emissions of current lettuce field farming were calculated for each field parcel using the DNDC model: a process-based model that incorporates a representation of the complex processes and interactions within a system, reliably predicting site-scale GHG fluxes under a range of different conditions (DNDC, 2018; Taft et al., 2019). Soil properties from the Harmonized World Soil Database and England's peaty soils map; regional climate data from the UK-MIDAS dataset; and management assumptions were incorporated in DNDC for calculating emissions (FAO, 2008; Met Office, 2024; Natural England, 2024). The results were aggregated by soil type and region, with impacts apportioned according to the proportional extent of mineral (80.5%) and peat (19.5%) soils used for lettuce production.

A detailed description of DNDC integration, input data and soil emissions methodology can be found in Supplementary Section C, and the results of soil emissions per soil type and per region for cultivating lettuce are available in Supplementary Section G.

Life cycle inventory analysis and impact assessment

Life cycle inventory (LCI) data for soil emissions and transportation are summarised in Table 2, complementing the core inventories available in Supplementary Section H. Additional details on LCI development and LCI tables are available in Supplementary Section H.

**Table 2:** LCI data for soil emissions and transportation for all scenarios (normalised to the functional unit). *These LCI values are in addition to core LCI's available in Supplementary Section H*.

Scenario	Process/activity	Units	Hyperlocal	Distribution centre	Field UK	UK mineral soil	UK peat soil
VF lettuce transportation	Delivery to Supermarket	kg km^−1^	0.066	26.85	-	-	-
Field lettuce transportation	Delivery to Supermarket (via distribution centres)	kg km^−1^	-	-	62.83	-	-
Field lettuce soil emissions	CO_2_ emissions avg.	kg CO_2_	-	-	-	0.001	0.26
	N_2_O emissions avg.	kg N_2_O	-	-	-	0.000046	0.000097
	CH_4_ emissions avg.	kg CH_4_	-	-	-	−0.0000015	−0.0000061

Ecoinvent v3.10 (Ecoinvent, 2022) served as the primary database for the LCI and emission factor data. Primary inventory data were sourced from Gargaro et al. (2025), with transportation and soil emissions recalculated to reflect UK-wide conditions. Foreground and background data were integrated using SimaPro v9.6 (Pre Sustainability, 2024) to compile and calculate the LCA.

The Life Cycle Impact Assessment (LCIA) applied the Product Environmental Footprint method (EF 3.1 in SimaPro v9.6). The LUNA model is underpinned by LCA principles, but its results are exclusively for climate change impacts. Consequently, this paper presents only the climate change (GHG) emission category for all scenarios.

### 2.4 Land use and energy modelling

The environmental impacts of alternative land sparing scenarios on land spared using VF is modelled using the LUNA tool (Hastings et al., in preparation). LUNA is underpinned by LCA methodology and quantifies direct and indirect GHG emissions arising from the conversion of land from its known 2025 baseline land use to alternative land sparing scenarios. Results are reported as cumulative GHG emissions per hectare through to 2050 (see Supplementary Section I for more information).

The scope of LUNA includes direct GHG emissions from site preparation, establishment, maintenance, and end-of-life processes, including product delivery and waste treatment (Hastings et al., in preparation). Changes in land carbon stocks, encompassing above- and below-ground biomass and SOC, are modelled throughout the life cycle. In addition, indirect emissions are captured through substitution credits for displaced fossil energy and wood products (Hastings et al., in preparation). Climate change outcomes are therefore driven by both land carbon dynamics and energy substitution.

However, in the VF scenarios, electricity use is treated as operationally decarbonised, reflecting procurement under a 100% renewable electricity supply contract, rather than electricity drawn directly from the average UK grid mix. As a result, renewable electricity generated on VF-enabled spared land is modelled as displacing average grid electricity, not VF electricity demand itself. This avoided emissions benchmark applied in LUNA is therefore defined relative to the current 2025 UK grid intensity, ensuring that displaced electricity reflects system wide decarbonisation benefits rather than changes internal to the VF system.

LUNA draws on the same soil data source used to model UK lettuce cultivation (Harmonized World Soil Database), which ensures consistency in baseline soil conditions across land-use transitions. In addition to life cycle GHG results, LUNA outputs projected changes in land carbon (which captures changes in soil and biomass carbon storage) and energy production (quantifies emissions avoided through renewable energy) to 2050. This dual framing reflects both terrestrial carbon dynamics and broader decarbonisation potential, enabling robust comparisons across diverse land sparing scenarios.

### 2.5 Full system integration

The following three ‘sub-systems’ were calculated as above:

• UK-wide VF system impacts for hyperlocal and distribution-centre scenarios
• UK-wide field farming and imported lettuce impacts
• Impacts of land-sparing scenarios

These results are combined to determine the full-system impacts of meeting UK lettuce demand through VF, including the effects of repurposing land spared from field production, and are compared against a business-as-usual scenario of field-based lettuce cultivation for the UK market. As land sparing scenarios are modelled from 2025 to 2050, impacts associated with both field farming and VF systems are assessed over the same 25-year period. Land-sparing benefits accumulate over time, with LUNA outputs increasing non-linearly on an annual basis, resulting in cumulative impacts by 2050. To quantify the benefits of land-sparing scenarios involving generated power, this study applies the average UK grid electricity carbon intensity for 2025 for avoided GHG emissions (Hastings et al., in preparation). Due to uncertainties with decarbonisation of the electricity system between 2025 and 2050, this analysis does not extend to modelling the prospective decarbonisation trajectory of the UK grid.

## 3 Results

### 3.1 Lettuce production stage impacts and land sparing potential

Land area spared

The maximum land area that could be spared within the UK is 4,000 ha, representing current open-field lettuce production, with protected cultivation (420 ha) excluded. Land sparing outside the UK is beyond the primary scope, but an indicative estimate suggests a further 4,490 ha could be spared abroad, based on the fact that 92% of UK lettuce imports originate from Spain, where production occupies 4,130 ha (FEPEX, 2022; Gobierno de Espana, 2021; HortoInfo, 2024). The land area needed for VF facilities in the UK is 590 ha for both hyperlocal and distribution centre scenarios, however, this is not included in the land sparing scenario calculations as these facilities are not placed on agricultural land or land spared by replacing field lettuce in this study. Methodological details are provided in Supplementary Section D.

Before further analysis, it is essential to contextualise UK lettuce demand in terms of per capita consumption, domestic production in open-field and protected environments, import volumes, and export flows. Full calculations underpinning these baseline estimates are provided in Supplementary Section A.

Production stage comparison

At the production stage, clear differences emerge between field-grown and vertically farmed lettuce in terms of climate change impacts, electricity demand and land requirements. A comparative summary of these national-scale production-stage results for UK field lettuce and both VF deployment scenarios is provided in Table 3.

**Table 3:** Comparative summary of production-stage climate change impacts, electricity demand and land requirements for UK field-grown lettuce and vertically farmed lettuce in the UK.

Process stage	Climate change (kg CO_2_ eq kg^−1^lettuce)	Electricity demand (kWh kg^−1^ lettuce)	Land demand (ha)
**UK field lettuce**	0.531	-	4,000
**VF lettuce (hyperlocal scenario)**	0.932	17.25	590
**VF lettuce (distribution centre scenario)**	0.935	17.25	590

Impact of field grown lettuce

**Fig. 3:**
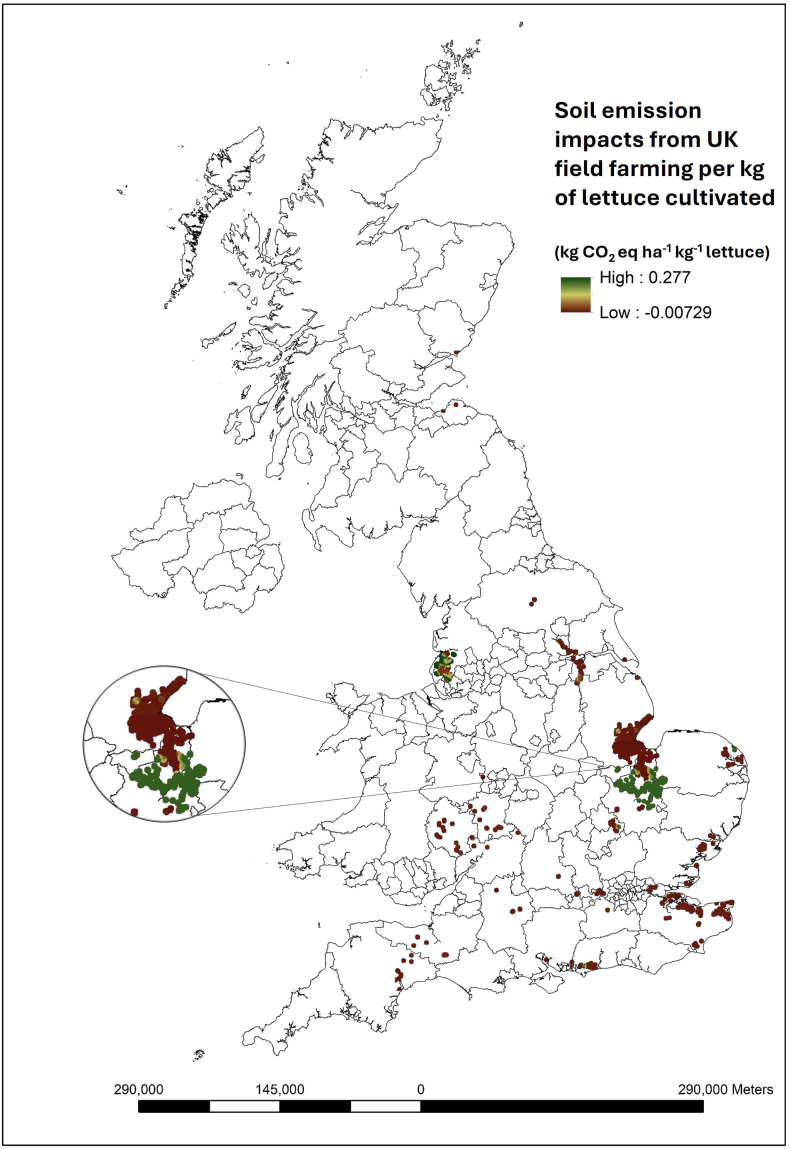
Spatially explicit map of UK lettuce cultivation areas with associated soil emissions per kg of lettuce cultivated (kg CO_2_ eq ha^−1^ kg^−1^ lettuce).

The average climate change impact of UK field-grown lettuce is 0.53 kg CO_2_ eq kg^−1^. Soil emissions were modelled across all UK lettuce cultivation areas, distinguishing between mineral and peat soils. Spatial variability in soil impacts is substantial (Fig. 3): 99.6% of lettuce production occurs in England, with 62% located in the Fens and East Anglia. Total impacts are similar for both mineral and peat soil types (0.532 kg CO_2_ eq kg^−1^ for mineral soils and 0.527 kg CO_2_ eq kg^−1^ for peat soils), but the composition of their impacts differs markedly (Table 4).

**Table 4:** LCA climate change impacts of field lettuce cultivation in the UK. Emissions are reported per kilogram of lettuce for the entire UK, with separate figures for cultivation (combined, mineral, peat) and transportation. Further results for all impact categories per kilogram of lettuce production are available in Gargaro et al. (2025).

Process stage	Climate change (kg CO_2_ eq kg^−1^lettuce)
**Cultivation (total UK)**	0.519
**Transport (total UK)**	0.0119
**Combined total UK**	0.531
**UK peat cultivation (without transport and soil emissions)**	0.270
**UK peat total (inc. transport and soil emissions)**	0.527
**UK mineral cultivation (without transport and soils)**	0.521
**UK mineral total (inc. transport and soil emissions)**	0.532

On peat soils, soil emissions contribute an average of 0.25 kg CO_2_ eq kg^−1^ lettuce, whereas mineral soils exhibit a small net sequestration effect (−0.0006 kg CO_2_ eq kg^−1^). Differences are driven primarily by SOC, which averages 39% in peat soils compared with 0.9% in mineral soils. Excluding soil emissions, peat-based cultivation impacts are 0.28 kg CO_2_ eq kg^−1^ lettuce, while mineral soil cultivation impacts remain essentially unchanged (0.52 kg CO_2_ eq kg^−1^ lettuce), reflecting peat's higher productivity but greater carbon sensitivity compared with mineral soils, which require higher inputs of fertilisers, pesticides, diesel, and irrigation.

At the national scale, transportation contributes only 2.2% of climate change impacts for UK lettuce production (Table 5). In contrast, imported lettuce from Spain has a lower cultivation footprint (0.4 kg CO_2_ eq kg^−1^ lettuce), but long-distance transport dominates the supply chain, accounting for ∼85% of climate change impacts. Spanish soils generally exhibit net carbon sequestration, offsetting 3–164% of cultivation emissions depending on the scenario (Gargaro et al., 2025). For further details on Spanish cultivation, including scenario-specific results see Supplementary Section D.

**Table 5:** LCA climate change impacts of lettuce cultivation in the UK, total for open field land area used for lettuce cultivation in the UK, and associated transportation of lettuce.

Process stage	Climate change (kg CO_2_ eq)
**Cultivation (total UK)**	4.57 × 10^7^
**Transport (total UK)**	1.04 × 10^6^
**Combined total (UK)**	4.67 × 10^7^

Impact of VF lettuce

Across both VF scenarios, electricity demand is the dominant hotspot for climate change impacts, accounting for 31% of total system emissions. Other major contributors include jute fibre production (21%), plastics used in cultivation and packaging (14%), hydrogen peroxide use (8%), and waste incineration (6%). Transportation contributes negligibly to overall climate change impacts (0.19% for hyperlocal and 0.55% for distribution-centre) (Table 6).

**Table 6:** LCA climate change impacts of lettuce cultivation in the UK for both hyperlocal (HL) and distribution-centre (DC) scenarios. Emissions are reported by total for the UK per kg of lettuce, with a breakdown of emissions for cultivation, and associated transportation of lettuce. Electricity demand per kg of lettuce is also presented.

Facility & Process stage	Climate change (kg CO_2_ eq kg^−1^lettuce)	Electricity demand (kWh kg^−1^ lettuce)
**UK-HL Cultivation**	0.93	17.25
**UK-HL Transport**	0.00177	-
**UK-HL Total**	0.932	-
**UK-DC Cultivation**	0.93	17.25
**UK-DC Transport**	0.0051	-
**UK-DC Total**	0.935	-

**Fig. 4:**
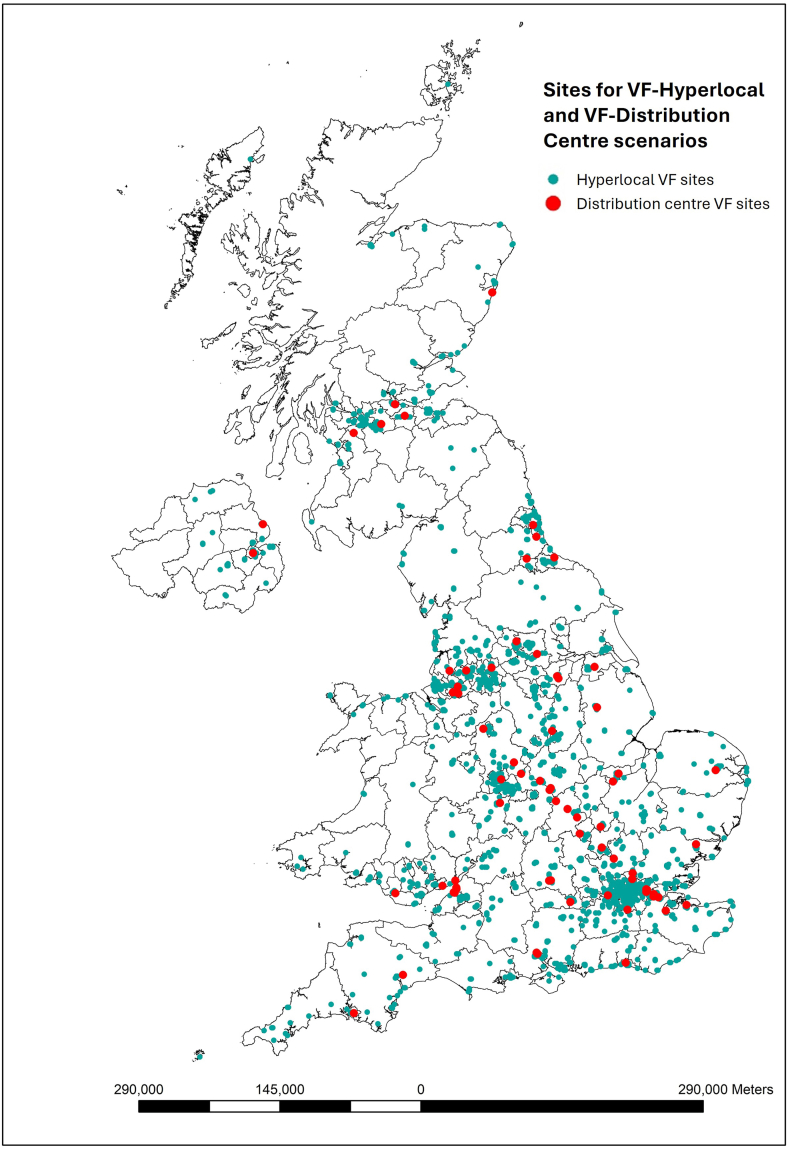
Spatially explicit map of the UK for siting of both hyperlocal (HL) scenario vertical farming facilities and distribution centre (DC) scenario vertical farming facilities.

When scaled to meet UK lettuce demand, the hyperlocal and distribution-centre scenarios differ by only 0.35% in climate change impacts. Due to facility siting assumptions, both hyperlocal and distribution-centre configurations indicate a potentially higher production capacity for VF lettuce than market demand, although for the analysis, facility production was constrained to UK demand (see Supplementary Section E). Table 7 summarises the impacts of national deployment of both scenarios, and Fig. 4 maps the spatial distribution of VF facilities for both scenarios.

**Table 7:** LCA impacts of lettuce cultivation in the UK for both hyperlocal (HL) and distribution-centre (DC) scenarios to fulfil the demand of the UK population (excluding overproduction in facilities).

Vertical farming scenario	Climate change (kg CO_2_ eq)	Electricity demand (KWh)	Land demand facilities (ha)
**HL total**	2.80 × 10^8^	5.19 × 10^9^	590
**DC total**	2.81 × 10^8^	5.19 × 10^9^	590

Although VF preserves the full land-sparing potential of open-field lettuce cultivation (4,000 ha), it is not land-independent. VF facilities occupy approximately 590 ha nationally. Climate change impacts are highly sensitive to electricity supply: with renewable electricity, VF emits 0.93 kg CO_2_ eq kg^−1^ lettuce, compared with 4.71 kg CO_2_ eq kg^−1^ under the current UK grid mix and 1.18 kg CO_2_ eq kg^−1^ under the projected 2050 grid Gargaro et al. (2025).

Supplying the UK lettuce market via VF requires an estimated 5,190 GW h of electricity annually. This demand provides the basis for assessing whether alternative uses of spared land could generate sufficient renewable energy to support VF at the national scale (Table 7).

### 3.2 Land sparing scenarios and system-level outcomes

This section evaluates land-use transitions, focusing on climate change impacts associated with shifts from current land use to alternative land sparing scenarios. While climate change impacts are a key metric, it represents only one dimension of system change; broader considerations such as biodiversity, water quality, and food security are addressed later in the paper.

LCA impacts overall

**Fig. 5:**
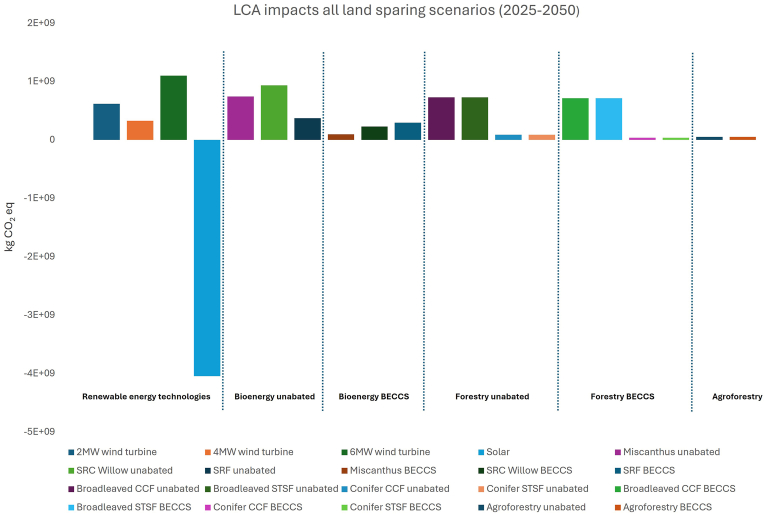
LCA climate change impacts (in kg CO_2_ eq) for all alternative land sparing scenarios evaluated on land spared from lettuce production in the UK (4,000 ha area); output for period 2025-2050 (25 years). Avoided emissions are benchmarked against the projected average UK grid electricity carbon intensity for 2025.

Over the 25-year assessment period, solar energy is the only land sparing scenario to deliver a net climate change benefit (Fig. 5 2), achieving cumulative negative climate change impacts of −4.05 × 10^10^ kg CO_2_ eq. This result is driven by high electricity output over a 30-year lifespan and substantial displacement of fossil-based grid electricity at current emissions rate. Although solar installations slightly reduce biomass inputs and SOC, these losses are outweighed by avoided energy emissions.

Wind energy scenarios exhibit net positive climate change impacts despite being renewable, due to embedded impacts from manufacturing, transport, and installation, combined with shorter operational lifespans. Among turbine sizes, the 4 MW option exhibits the lowest climate change impacts (3.28 × 10^8^ kg CO_2_ eq), while 2 MW and 6 MW turbines exhibit higher climate change impacts due to non-linear scaling of embodied impacts relative to energy output.

Across bioenergy scenarios, BECCS substantially reduces net climate change impacts relative to unabated systems. *Miscanthus* and SRC willow impacts are reduced by 87% and 75%, respectively, while coniferous forestry with BECCS achieves a reduction of approximately 60%. Agroforestry systems exhibit low climate change impacts but limited mitigation potential, as low tree cover results in insufficient biomass for meaningful carbon capture.

Peat soils exert a dominant influence on LUC outcomes. Due to their high SOC content and sensitivity to disturbance, carbon losses from peat soils outweigh gains from vegetation growth or energy substitution across all land sparing scenarios. This effect represents a critical constraint on land-based mitigation and is examined further in subsequent sections.

System-level impacts

**Fig. 6:**
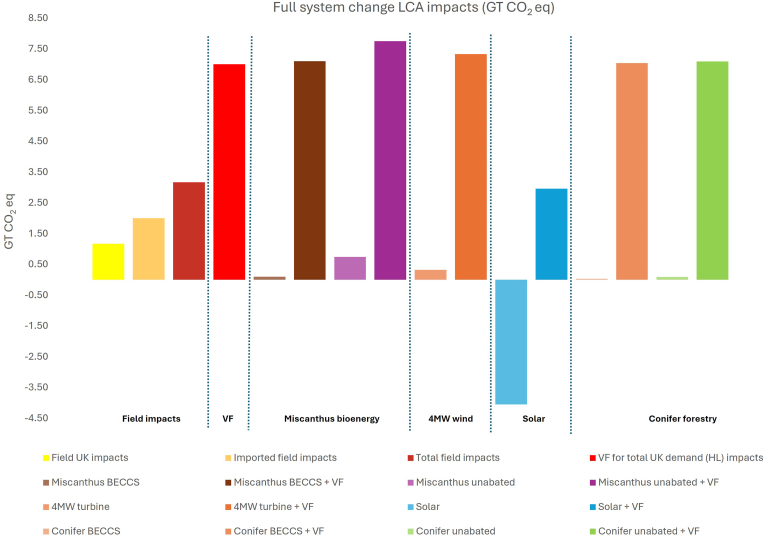
Full system LCA climate change impacts (in GT CO_2_ eq) for six selected land sparing scenarios (Conifer forestry (BECCS and unabated), Miscanthus (BECCS and unabated), solar energy, and 4 MW wind turbines) individually, and combined with vertical farming impact. UK field grown and imported lettuce, total field impacts, and total UK vertical farming impacts are also presented; output for period 2025-2050 (25 years). Avoided emissions are benchmarked against the projected average UK grid electricity carbon intensity for 2025.

From the 20 land sparing scenarios assessed, the highest-ranking option within each category was selected for comparison: conifer forestry (BECCS and unabated), *Miscanthus* (BECCS and unabated), solar energy, and 4 MW wind turbines (Fig. 6).

Consistent with the land-sparing analysis, solar energy deployed on spared land is the only scenario to deliver net negative climate change impacts, making it the most effective climate change mitigation option. Baseline climate change impacts from UK field-grown lettuce total 1.17 Gt CO_2_ eq, with imports contributing a further 2.00 Gt CO_2_ eq, resulting in 3.17 Gt CO_2_ eq for current UK lettuce procurement. Supplying the same demand entirely through VF under the hyperlocal scenario results in 7.01 Gt CO_2_ eq, reflecting the high electricity demand of VF systems.

Repurposing spared land for solar energy yields −4.05 Gt CO_2_ eq, offsetting VFs climate change impacts and reducing the combined total to 2.96 Gt CO_2_ eq, which is 0.21 Gt CO_2_ eq below the field-farming baseline. In contrast, deployment of 4 MW wind turbines on spared land adds 0.33 Gt CO_2_ eq, increasing total system climate change impacts to 7.33 Gt CO_2_ eq.

Forestry scenarios perform better than wind, with conifer systems contributing 0.04–0.09 Gt CO_2_ eq, resulting in total climate change impacts of 7.04–7.10 Gt CO_2_ eq. This performance reflects rapid biomass accumulation, long-lived wood products, and limited soil disturbance, which enhance substitution credits and carbon storage.

Overall, only the combination of VF with solar energy on spared land achieves a total climate change impact below the field lettuce baseline. All other land-use options provide partial mitigation but do not deliver net system-wide GHG reductions relative to conventional field production.

### 3.3 Key land and energy perspectives

Land carbon impacts

**Fig. 7:**
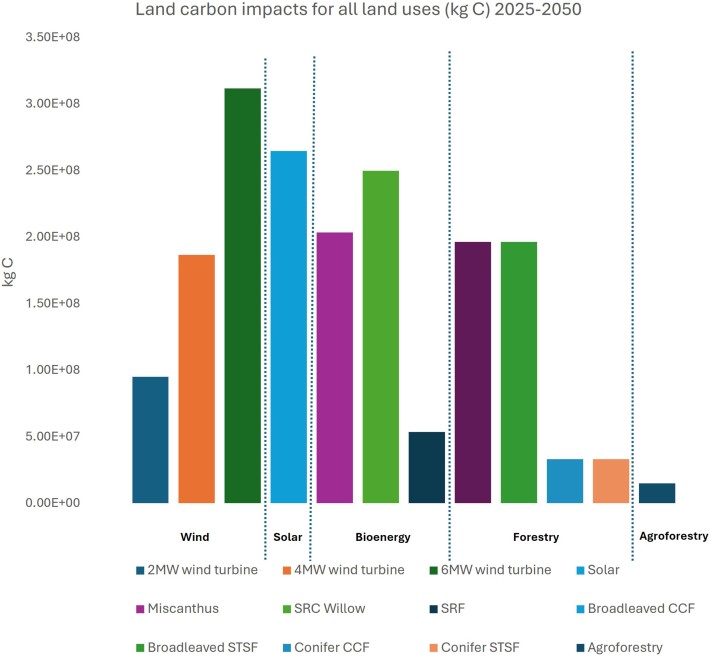
Land carbon impacts (in kg C) for all alternative land sparing scenarios evaluated on land spared from lettuce production in the UK (4,000 ha area), including mineral and peat soils in their national proportions; (land carbon impacts are the same for unabated and BECCS scenarios); output for period 2025-2050 (25 years). Lower land carbon values correspond to greater sequestration and therefore stronger climate benefits, while higher values indicate net carbon loss.

Agroforestry exhibits the lowest land carbon impact (Fig. 7 3), at 1.50 × 10^7^ kg C, reflecting minimal soil disturbance and continuous carbon inputs from integrated tree–crop systems; however, the land-carbon result relates only to the tree strips and does not include emissions from the cropped proportion of the system. Coniferous forestry systems (CCF and STSF) also perform well, each recording 3.31 × 10^7^ kg C, supported by continuous canopy cover and longer rotations that maintain SOC. Short-rotation forestry follows at 5.37 × 10^7^ kg C, with sequestration constrained by frequent harvesting.

Intermediate impacts are observed for 2 MW wind turbines (9.51 × 10^7^ kg C), while perennial bioenergy crops show substantially higher land carbon losses, with *Miscanthus* and SRC willow at 2.04 × 10^8^ kg C and 2.50 × 10^8^ kg C, respectively, reflecting soil disturbance during establishment. The highest land carbon impacts occur for non-biological renewable energy systems, solar installations (2.65 × 10^8^ kg C) and 6 MW wind turbines (3.12 × 10^8^ kg C), driven by extensive ground preparation and infrastructure footprints.

**Fig. 8:**
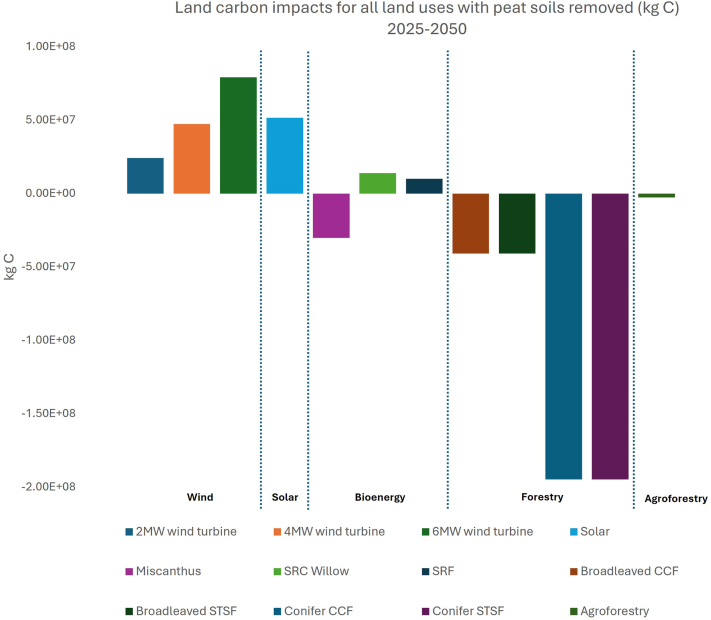
Land carbon impacts (in kg C) for all alternative land sparing scenarios evaluated on land spared from lettuce production on mineral soils only in the UK; (land carbon impacts are the same for unabated and BECCS scenarios); output for period 2025-2050 (25 years). Lower land carbon values correspond to greater sequestration and therefore stronger climate benefits, while higher values indicate net carbon loss.

To isolate mineral soil responses, peat soils (SOC >0.2 (Hunt et al., 2025)) were removed from the analysis (Fig. 8). This adjustment reveals markedly different outcomes, with several biological land uses transitioning from net emissions to net sequestration. Broadleaved forestry systems (CCF and STSF) shift from +1.96 × 10^8^ kg C to −4.08 × 10^7^ kg C, while *Miscanthus* moves to −3.02 × 10^7^ kg C, reflecting biomass accumulation and SOC recovery on mineral soils. Agroforestry remains near carbon neutral (−2.69 × 10^6^ kg C), consistent with its low tree density and limited contribution to SOC.

In contrast, wind and solar installations remain net land carbon emitters, even with peat excluded. For example, 6 MW wind turbines decline from +3.12 × 10^8^kg C to +7.92 × 10^7^kg C, and solar from +2.65 × 10^8^kg C to +5.16 × 10^7^ kg C, indicating that construction-related soil disturbance prevents net sequestration. Full results for peat-excluded scenarios are provided in Supplementary Section K.

In addition to isolating mineral-soil responses, we also explored the alternative case in which the peat soil area is restored rather than converted to alternative land uses. Applying global average emission factors for restored peatland resulted in far lower climate change impacts (1.45 × 10^5^ kg CO_2_ eq) compared with conversion to alternative land sparing scenarios modelled in this study (average impact of 1.54 × 10^8^ kg CO_2_ eq). This contrast highlights the extent to which peatland recovery represents a substantially lower-impact pathway than repurposing peat soils for new land uses (see Supplementary Section L for global average emission factors for restored peatland).

Energy production

**Fig. 9:**
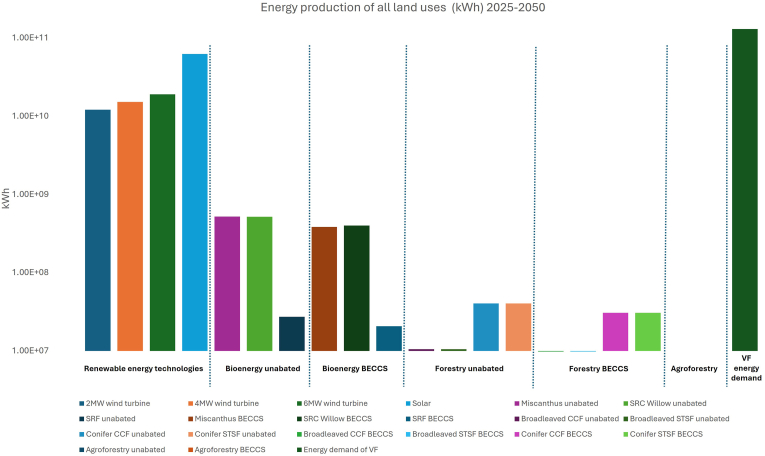
Energy Production (in kWh) for all alternative land sparing scenarios evaluated on land spared from lettuce production in the UK (4,000 ha area); output for period 2025-2050 (25 years). Agroforestry (unabated and BECCS) does not produce energy (hence the gap in the graph). The logarithmic Y-axis starts at 1 × 10^7^.

Solar installations dominate energy production, delivering 6.23 × 10^10^ kWh over 25 years, far exceeding other options (Fig. 9 4). Wind energy outputs scale with turbine size: 6 MW turbines (1.90 × 10^10^ kWh), 4 MW (1.52 × 10^10^ kWh), and 2 MW (1.21 × 10^10^ kWh), highlighting the high energy density of non-biological renewables compared to biomass systems.

Bioenergy crops yield substantially less energy. *Miscanthus* and SRC willow produce 5.22 × 10^8^ kWh and 5.18 × 10^8^ kWh when unabated, falling to 3.85 × 10^8^ kWh and 4.00 × 10^8^ kWh respectively with BECCS, reflecting carbon capture-related energy penalties. Forestry scenario energy outputs are minimal as they are grown predominantly for timber: broadleaved CCF and STSF deliver 1.05 × 10^7^ kWh (8.01 × 10^6^ kWh with BECCS), coniferous systems 4.04 × 10^7^ kWh (3.07 × 10^7^ kWh with BECCS), while agroforestry contributes no measurable energy, consistent with its design as a multifunctional land use rather than an energy system.

When comparing energy outputs with VF electricity demand, supplying all 300,800 t of UK lettuce demand requires 1.30 × 10^11^ kWh; the 88,000 t corresponding to UK spared land requires 3.80 × 10^10^ kWh. Solar alone can meet 164% of UK spared-land VF demand and 48% of full UK lettuce VF demand. 6 MW wind meets ∼50% of spared-land demand and 15% of full demand. Bioenergy crops, even with BECCS, contribute less than 2% of spared-land VF demand.

## 4 Discussion

This study provides a novel, full-system assessment of UK lettuce production, comparing conventional field cultivation with national-scale VF and repurposing of spared land to improve environmental outcomes. By integrating LCA with spatially explicit land-sparing modelling, the analysis moves beyond production-stage comparisons and evaluates how a national transition from field-grown lettuce to VF could reshape environmental outcomes across the wider land-use system. Given the UK's intense competition for land, arising from simultaneous targets for food production, renewable energy, biodiversity recovery, and climate mitigation, understanding how VF-enabled land sparing interacts with these demands is particularly important.

Although the analysis presented here is scenario-based, VF is no longer a theoretical concept. Commercial VF facilities are already operating in the UK and internationally, and despite economic and technical challenges, the sector continues to expand (GVH, 2025; GVR, 2025; Namkung, 2022). As further VF facilities are developed and the sector continues to grow, any potential environmental impacts, both positive and negative, will begin to materialise. The sector has experienced periods of rapid investment alongside boom-and-bust cycles of VF companies, creating an urgent need to understand these impacts now before deployment accelerates further (Bullock, 2025; Harvey, 2021; UCL, 2025). Quantifying these impacts is therefore essential for understanding the real-world implications of VF adoption and for informing how such systems should be integrated into wider land-use planning, especially as governments and research institutions increase their involvement through funding programmes and policy-adjacent initiatives (UKRI, 2023, 2024b, 2024a).

Scenario results represent steady-state outcomes and do not simulate transitional dynamics or time-dependent processes, such as woodland growth trajectories, so temporal variation in impacts is not captured. The 25-year modelling horizon reflects short-to medium-term outcomes and does not include the longer-term carbon dynamics of slower-acting land-use transitions. Much of the carbon benefit of woodland creation and longer-rotation systems occurs beyond 25 years, meaning forestry pathways may appear less effective here than they would over longer timescales (Forest Research, 2023a; Wang et al., 2025). As the timeframe is short, initial disturbance effects have a proportionally larger influence on results. As such, if these scenarios were modelled over longer time horizons, the relative performance of some land-use pathways, particularly forestry, may differ, and wind land-impact reduces on repowering as the existing roads and hardstanding are reused.

A key finding however is the trade-off between VF's very high land use efficiency and its relatively high per-kg climate change impacts, driven primarily by electricity demand. While VF achieves substantially higher yields than field systems, this does not automatically translate into lower production stage impacts.

Instead, VF's environmental benefits stem from the land it can spare. With yields ∼24 times higher than field-grown lettuce, VF can release up to 4,000 ha of agricultural land in the UK. This land-sparing potential underpins the evaluation of alternative land uses and their influence on system-level outcomes, which depend on whether objectives prioritise GHG mitigation, land carbon protection, biodiversity, or wider ecosystem services.

### 4.1 Vertical farming: land efficiency vs emissions

VF offers a significant increase in land-use efficiency compared to conventional field systems. Achieving yields of ∼97 kg lettuce m^−2^ is consistent with reported values of 95 to 101 kg m^−2^ (Blom et al., 2022; Perez, 2014; Touliatos et al., 2016). Meeting the UK's annual lettuce demand would require 172 ha of VF growing space, rising to 590 ha once the full operational footprint are included (e.g. seed preparation area, harvesting rooms etc.), resulting in a net land sparing of 3,410 ha. Although additional land sparing outside the UK (∼4,490 ha) is possible, this lies beyond the scope of this study.

However, this land efficiency comes with a clear trade-off. VF's per-kg climate change impacts exceed those of UK-field grown lettuce, driven primarily by high electricity demand. Even under a 100% renewable electricity scenario (as modelled in this paper), VF climate change impacts remain ∼75% higher than field production (0.93 vs 0.53 kg CO_2_ eq kg^−1^ lettuce). When modelled using current UK grid mix or projected 2050 grid, impacts increase substantially, up to nine times those of field production (Gargaro et al., 2025). Electricity is the dominant hotspot, accounting for 31% of climate change impacts under renewable supply and 86% under current grid electricity, largely due to artificial lighting and climatic control requirements (Gargaro et al., 2025). These findings align with Sandison et al. (2023) where energy use accounted for 91% of VF's carbon footprint, and Casey et al. (2022) who reported climate change impacts of 1.5–8.9 kg CO_2_ kg^−1^ produce using grid electricity, dropping to 0.33–0.56 kg CO_2_ with renewables. These findings underscore the urgent need for decarbonising energy supply for VF via renewable energy integration.

The electricity demand assumed for VF in this analysis reflects current commercial systems rather than prospective technological trajectories. Over a 25-year time horizon, further improvements in production technologies, particularly in LED efficiency (lighting consumes ∼56% of this vertical farm's electricity demand), are likely and would be expected to reduce the electricity intensity of VF and, in turn, its production-stage climate change impacts (Gargaro et al., 2024; Paucek et al., 2020; Aborujilah, 2025). Such efficiency gains would narrow the gap between VF and field cultivation at the production stage, but would not materially change the overall interpretation of the results unless electricity demand were reduced substantially, to the point where VF production-stage impacts fall to a comparable range or below those of field farming.

Transportation impacts were negligible, contributing less than 1% of total climate change impacts across both VF scenarios, highlighting that proximity to population centres does not materially reduce VF's climate change impacts, and that transport impacts are not a key factor in determining the environmental viability of VF on a national scale. This shows that whether VF is hyperlocal or distributed has virtually no effect on its climate change impact. Siting decisions should instead focus on factors such as low-carbon electricity availability, land suitability, cost efficiency, and opportunities for local employment rather than minimising transport distance.

This combination of high land efficiency and energy intensity shows that VF's production footprint cannot be assessed in isolation. Its environmental value depends on how the 4,000 ha of spared field land is repurposed, with system-level outcomes determined by the performance of alternative land uses rather than production-stage emissions alone. This interaction provides the rationale for evaluating VF within a full system land sparing framework.

### 4.2 Land sparing as the mechanism driving system outcomes

Vertical farming shifts the locus of climate change impacts from the production facility to the land made available once field-based lettuce cultivation is removed. In this analysis, relocating lettuce production indoors frees the full 4,000 ha currently used for UK field production, creating opportunities for LUCs that would not otherwise occur. This land-sparing effect is the primary mechanism through which VF influences wider environmental outcomes, with system-level climate change impacts determined less by operational emissions than by how the freed land is subsequently used. The following sections therefore examine alternative land-use pathways and their implications for climate change impacts, land carbon dynamics, and broader ecosystem services.

These findings align with and extend previous work highlighting land sparing as a climate-mitigation strategy, while emphasising that its benefits are strongly conditional on how spared land is used. Lamb et al. (2016), using the UK as a case study, showed that land sparing enabled by agricultural efficiency gains could substantially offset GHG emissions, but also demonstrated that mitigation outcomes differ markedly between forestry, bioenergy and other land-use pathways. Our results reinforce this conclusion by showing that identical areas of spared land can generate contrasting climate outcomes depending on whether they are allocated to renewable energy, bioenergy or forest systems, with some pathways providing limited or even adverse mitigation benefits.

Similarly, UK-scale assessments such as Vetter et al. (2021) demonstrate that achieving net-zero agricultural outcomes depends critically on land-use change, particularly through peatland restoration, afforestation, and changes in agricultural management. Our results are consistent with this conclusion and extend it by showing that, when agricultural land is made available through changes in food production systems, climate outcomes depend strongly on how that land is subsequently allocated, and that inappropriate land-use choices can undermine expected mitigation benefits.

### 4.3 GHG mitigation: solar as the optimal pathway

Among the land uses modelled, solar energy emerges as the most effective pathway for climate change mitigation. Solar avoids 4.05 Gt CO_2_ eq over 25 years, primarily through displacement of fossil-based grid electricity. Although VF has higher climate change impacts than field systems in isolation (7.01 vs 3.17 Gt CO_2_ eq), pairing VF with solar reduces total system climate change impacts to 2.96 Gt CO_2_ eq. However, even with the high-efficiency panels (25%) modelled in LUNA, solar on all the spared land cannot generate enough power to meet the full UK lettuce demand if it were produced entirely in VF.

Solar's mitigation strength reflects its high energy density and conversion efficiency relative to alternative land uses. It produces up to 3.3 times more energy per hectare than 6 MW wind turbines and far exceeds unabated biomass (<120 times) and BECCS (<162 times) in the areas of land spared. UK planning-based estimates similarly show solar delivers several times more energy per hectare than wind (∼376–1506 vs ∼62–158 MWh ha^−1^ yr^−1^) (Gillet, 2022). Findings by Mallins (2014) support these outcomes also, highlighting the high energy density of solar compared with wind (∼1,000,000 vs ∼40,000 MJ ha^−1^), and BW (2016) finding solar achieves much higher energy production than bioenergy from woodlands (482 vs 12 MWh ha^−1^ yr^−1^). This spatial advantage translates into lower lifecycle climate change impacts: solar produces roughly one-third the climate change impacts of onshore wind per unit of electricity (46 vs 126 g CO_2_e kWh^−1^) (NREL, 2012). These differences are driven by conversion efficiency, with solar panels converting ∼25% of incoming energy to electricity in LUNA, compared with 4–6% for photosynthesis (X.-G. Zhu et al., 2008). These findings relate only to climate change impacts; other life-cycle impact categories, including resource use, may differ and were outside the scope of this analysis.

In contrast, wind does not deliver a net climate change benefit on spared land. All wind scenarios result in net positive climate change impacts due to high embodied carbon in construction materials, which scales sharply with turbine size (Smoucha and Fitzpatrick, 2016; Tri and Johnson, 2023). Although energy output remains substantial for 6 MW turbines (1.90 × 10^10^ kWh), lower energy density limits fossil-fuel displacement, and even the most favourable wind configuration (4 MW turbines, 0.33 GT CO_2_ eq) increases system climate change impacts relative to VF alone.

BECCS improves performance relative to unabated bioenergy but remains insufficient to offset VF's operational climate change impacts. Low energy output and the energy penalty of carbon capture constrain mitigation potential (Fajardy et al., 2019; Harrison and MacDonald, 2024; ScotGov, 2024), leaving combined VF–BECCS systems above the field-farming baseline despite low climate change impacts for BECCS’ land sparing scenarios (0.1 Gt CO_2_ eq).

Overall, these results show that solar is the only land-use pathway capable of offsetting VF's operational climate change impacts at the national scale.^5^ However, when objectives shift from climate change mitigation to land-carbon protection, relative performance is reshaped by soil carbon responses rather than energy output.

Nonetheless, before comparing land-carbon outcomes, it is important to recognise that the climate change mitigation potentials reported reflect only the LCA system boundary and do not account for wider energy-system characteristics. For example, solar and wind generation are inherently intermittent, whereas biomass feedstocks are storable and dispatchable, allowing them to provide energy on demand (Delarue and Morris, 2015; FAO, 2022). These system-integration attributes were not modelled in the LUNA framework but are relevant when interpreting how land-sparing options may interact with real-world energy-system needs.

Interpretation of these avoided-emissions outcomes also depend on assumptions regarding the electricity system against which substitution is assessed. This analysis applies a static avoided-emissions benchmark based on the average UK grid intensity in 2025, which is held constant across the 2025–2050 assessment period. This approach avoids introducing additional uncertainty associated with forecasting long-term changes in the electricity system. However, as the UK grid continues to decarbonise over time, the amount of emissions displaced per unit of solar or wind generation would be expected to decline. As a result, avoided-emissions estimates associated with renewable deployment on spared land are likely to be lower in later years than those implied by a fixed 2025 benchmark. Importantly, this affects the magnitude of avoided emissions rather than their direction, and does not change the relative ranking of land-use pathways or the central finding that climate outcomes are highly sensitive to how spared land is repurposed. The comparative conclusions drawn from the land-sparing scenarios therefore remain robust under future grid decarbonisation.

It is also important to note that infrastructure-related emissions are included for alternative land-use pathways modelled on VF-enabled spared land but are excluded from the VF production system itself, reflecting both the limited availability of robust primary data for VF facility construction and the analytical focus on operational GHG emissions. This asymmetry introduces a bias in system-level comparisons and likely results in a conservative outcome, leading to an underestimation of the total climate change impacts associated with VF-enabled land sparing; however, given that VF climate change impacts over the 25-year assessment period are dominated by operational electricity demand, inclusion of VF infrastructure emissions would be unlikely to substantially change overall outcomes or the relative ranking of land-sparing pathways, although absolute impacts could increase.

### 4.4 Land-carbon protection: forestry and agroforestry

When the objective shifts from climate change mitigation to land-carbon protection, the relative performance of land uses changes. Outcomes are driven primarily by soil carbon responses to vegetation cover, soil disturbance, and long-term biomass dynamics, favouring biological systems that supply continuous organic inputs and maintain vegetation cover. In contrast, solar and wind exhibit higher land-carbon impacts due to construction disturbance and the absence of biological inputs to offset SOC losses (Armstrong et al., 2014; Carvalho et al., 2024; Pekkan et al., 2021).

Among biological systems, agroforestry exhibits the lowest land-carbon impact (1.50 × 10^7^ kg C over 25 years). Minimal soil disturbance and sustained organic inputs allow silvoarable systems to sequester up to 8 t CO_2_ ha^−1^ yr^−1^ (Woodland Trust, 2022a; Zhu et al., 2020). Forestry systems also perform strongly (3.31 × 10^7^ kg C). Trees accumulate carbon above- and belowground, with litter and root turnover increasing SOC, especially on low-carbon soils (Cunningham, 2024; Lukac, 2012; Wang et al., 2024). Sequestration patterns in forestry are shaped by rotation regimes: STSF systems accumulate carbon rapidly but show post-harvest losses, while CCF systems accumulate carbon more slowly but consistently (Hastings et al., in preparation). Collectively, forestry systems maintain SOC by limiting disturbance and supplying sustained organic matter input, making them the most favourable options under a land-carbon objective.

Perennial energy crops such as *Miscanthus* and SRC willow deliver intermediate outcomes, (2.04 × 10^8^ kg C and 2.50 × 10^8^ kg C), reflecting steady biomass inputs and extensive root systems, leveraging their fast-growing, low-input and high-yielding nature (Bottoms, 2012; DESNZ, 2023; Rowe et al., 2016). However, sequestration varies spatially; *Miscanthus* can sequester 0.7–2.2 t C ha^−1^ yr^−1^ under optimal conditions but may exhibit net losses in cooler regions (Abdalla et al., 2025; McCalmont et al., 2017).

Solar and wind generate the highest land-carbon impacts among the land uses assessed (2.65 × 10^8^kg C and 3.12 × 10^8^ kg C respectively), driven primarily by construction disturbance and reduced vegetation cover. For solar, SOC losses arise during site preparation through grading and vegetation removal, which releases stored carbon, and persist during operation due to panel shading and routine mowing that limit biomass inputs and SOC accumulation (Carvalho et al., 2024; Green, 2025; He, 2025; Krasner et al., 2025; Rangarajan et al., 2022; Schmidt et al., 2017). Although mitigation measures such as agrivoltaics or wildflower corridors may partially offset SOC losses, achieving sufficient irradiance (>65%) under panels remains challenging (ADAS, 2023; Carvalho et al., 2024; Jamil and Pearce, 2025; Scholten et al., 2025; van de Ven et al., 2021). Wind energy produces similarly high land-carbon impacts due to the spatial extent of construction disturbance. Turbine foundations, access roads and cabling disturb approximately 15–18% of field sites (Hastings et al., in preparation), with empirical studies reporting sustained vegetation losses and SOC reductions following installation (Armstrong et al., 2014; Li et al., 2024; Pekkan et al., 2021).

Overall, biological land uses provide the strongest pathways for SOC protection, with agroforestry ranking highest in this analysis. However, the relative performance of these land uses is overshadowed when high-SOC soils are considered, as peatland responses dominate land-carbon outcomes irrespective of land use-type.

### 4.5 Peat soils: high carbon constraint

Peat soils contain exceptionally high SOC stocks and are highly sensitive to disturbance, explaining their dominant influence on land-carbon outcomes (Chiti et al., 2024). Across all scenarios modelled, LUC on peat results in net climate change impacts. When peat is excluded and we evaluate the impact on mineral soils only, many biological systems shift to net sequestration, while renewables remain net emitters due to a lack of biological carbon inputs.

The disproportionality is further highlighted when we consider the relative distribution of soils cultivated. Although mineral soils account for approximately 80.5% of UK lettuce production land, peat soils, representing only 19.5%, exert a disproportionately large influence on national-scale outcomes. Their combination of high SOC stocks and strong sensitivity to disturbance means that emissions from this small proportion of land dominate aggregate results, effectively overshadowing the behaviour of mineral soils. This contrast highlights how even a relatively small peat area can mask the behaviour of mineral soils and dominate system-level climate change outcomes, and why understanding peat-soil impacts is essential when interpreting national-scale land-use transitions.

Overall, LUC on peat soils should be avoided. European peat stores ∼578 t C ha^−1^ versus ∼108 t C ha^−1^ for mineral soils, meaning identical land-use transitions generate orders of magnitude higher emissions on peat (Chiti et al., 2024). This pattern is clearly reflected in the modelling results in our present study.

Applying global average emission factors for restored peatlands produced substantially lower climate change impacts than conversion to alternative land sparing scenarios modelled (∼1.45 × 10^5^ vs ∼1.54 × 10^8^ kg CO_2_ eq). However, peat restoration carries risks; poor hydrological management can increase methane and nitrous oxide emissions, and misapplied burning can exacerbate degradation (IUCN, 2024; Noble et al., 2025).

### 4.6 Biodiversity and ecosystem services

While climate change mitigation and land-carbon outcomes are central metrics, they represent only one dimension of environmental performance. Although solar emerges as most effective for life-cycle climate change impact reduction, it provides comparatively limited ecosystem service benefits.

Forestry, bioenergy and peatland restoration deliver broader ecosystem services. Forestry enhances habitat complexity, increasing species richness by 40–60% relative to arable land, while also regulating water flows and reducing flood peaks by up to 20% (Beesley et al., 2025; Forest Research, 2023b; Woodland Trust, 2022b, 2025). These benefits are highly site-dependent; inappropriate species selection or planting on peat can undermine both carbon and ecological outcomes (Ashwood et al., 2015; Sloan et al., 2018). The principle of “right tree, right place” is critical to avoid unintended consequences.

Agroforestry offers even broader ecosystem benefits by improving soil structure, nutrient cycling and water infiltration, reducing runoff and enhancing biodiversity (Woodland Trust, 2022a; Zhu et al., 2020). Hedgerows and riparian buffers further enhance connectivity by providing corridors for wildlife, yet uptake remains limited by cost, lack of advice and uncertain markets for tree crops (Woodland Trust, 2022a).

Though bioenergy crops do offer moderate soil and climate change benefits, monoculture practices can limit biodiversity; *Miscanthus* for example supports higher pollinator abundance than cereals but remains less biodiverse than semi-natural habitats (Hodgson et al., 2024). In contrast, SRC willow is typically planted as mixed multi-genotype plantations and has well documented biodiversity advantages, supporting higher plant and animal species richness than arable land (Kwapong, 2024). All perennial crops reduce runoff and soil erosion, and increase SOC in mineral soils (Singh, 2025).

Finally, peatland restoration provides substantial ecosystem benefits beyond climate change mitigation, providing critical water regulation benefits, improving biodiversity by supporting rare species, and flood mitigation, reducing flood peaks ten-fold compared to degraded peat, though outcomes depend on careful restoration design (Brotherton, 2025; Brown, 2024; Holden et al., 2008; UoL, 2020).

### 4.7 Multifunctional landscapes and policy

Environmental performance of land-use transitions is context-dependent. Solar is most effective for climate change mitigation, forestry ranks highest for land-carbon protection, and biological systems deliver the strongest biodiversity and ecosystem-service outcomes. Peat soils remain an overriding constraint across all land-use transitions, elevating climate change impacts relative to mineral soils. Given competing land demands in the UK, no single land-use option can satisfy all objectives, making trade-offs unavoidable and requiring integrated strategies that can deliver on multiple targets (DEFRA, 2025b). This reinforces the principle of spatial targeting- deploying the right land use in the right location to maximise environmental benefits.

Although this analysis adopts a primarily GHG-centric lens, broadening the perspective highlights additional priorities including food security, resilience, water efficiency and biodiversity restoration. VF offers resilience benefits through high yields per unit area, controlled production, and reduced exposure to climate shocks and import dependence, but its high energy demand remains a critical constraint (Gargaro et al., 2024, 2025), as does the cost of VF produce (Moghimi and Asiabanpour, 2023; Tasgal, 2019). As broader objectives are considered, attention shifts from identifying a single optimal land use to designing integrated multifunctional land use strategies that deliver multiple outcomes simultaneously.

This shift is increasingly reflected in UK policy, which identifies multifunctionality as essential for meeting Net Zero, biodiversity, and domestic food production targets (DEFRA, 2025b, 2025c). The Royal Society similarly advocates for integrated land use strategies that deliver multiple benefits, estimating that up to 4.4 million ha (18% of UK land) may need to change use by 2050, underscoring the scale of coordinated land use transformation required (Royal Society, 2023).

The results of this study illustrate how such integration might operate in practice. While 61% of spared lettuce land would be required for solar to offset VF climate change impacts associated with UK field production (88 million kg lettuce yr^−1^), remaining land could support peatland restoration, woodland creation and agroforestry, jointly delivering climate change mitigation, biodiversity recovery and water regulation- objectives no single land-use option can achieve alone.

Delivering these outcomes requires locally tailored governance that reflects regional needs and constraints (FFCC, 2023). Land-use priorities vary by soil type, geography and socio-economic context. For example, a multifunctional strategy for East Anglia, dominated by peat soils, will differ from one for upland Wales or peri-urban London. This is reflected in the introduction of Local Nature Recovery Strategies (LNRS) under the Environment Act, which embed biodiversity and ecosystem services into spatial planning at the local authority level (DEFRA, 2023). Pilot studies at local authority level could build on LNRS principles to explore integrated scenarios that account for multiple environmental outcomes.

Ultimately, there is not a binary choice between VF and field farming, or between solar and forestry. It is a balancing act, optimising land for multiple outcomes rather than pursuing a single metric in isolation. Multifunctional landscapes, grounded in local knowledge and supported by adaptive governance, may therefore offer the most promising pathway for sustainable land management in the UK.

## 5 Recommendations for future research

Future research should extend this analysis to other crops and geographical contexts to assess whether the land-sparing potential of VF observed for UK lettuce applies more broadly across the horticultural sector. Applying the methodology to crops with different growth cycles, yields, and input requirements would clarify the extent to which results are crop-specific and how crop choice influences environmental outcomes. Replicating the analysis in regions with contrasting climates, land constraints, and energy systems would further clarify the role contextual factors have on environmental outcomes, including potential indirect land-use effects.

Future research should also move beyond the straight one-to-one land substitution applied in this study, and explore more realistic, system-wide patterns of land reallocation. Although replacing field lettuce with solar produces the strongest climate change impact reductions, prime agricultural land (ALC grade 1) is unlikely to be converted to solar given its value for food production (Natural England, 2021). A more realistic pathway is conversion to other food crops, such as wheat, which is typically grown on lower-grade land (ALC grade 3a). Higher-grade soils produce higher yields, so shifting wheat to ALC grade 1 land could reduce national wheat land demand, freeing more lower-grade land for alternative uses. Modelling these dynamic, multi-crop, multi-grade land use transitions would provide a more holistic understanding of national scale LUC.

Further work should also explore how improvements in VF productivity and energy efficiency affect trade-offs between food supply, land sparing, and climate change impacts. Modelling alternative yield and energy scenarios would provide a more nuanced assessment of VF under different technological and energy system assumptions. Crop-specific LCAs will be particularly important, as results for leafy greens may not translate to other horticultural crops.

Subsequent analyses should also examine land sparing scenarios without accounting for avoided fossil-fuel substitution from renewable energy generation, which can obscure impacts on soil carbon, biodiversity, and other ecosystem services. Expanding the LCA beyond climate change impacts to include additional environmental indicators would further strengthen system-level evaluation. Finally, improved spatial data, inclusion of construction impacts, and consideration of economic feasibility and social acceptance are needed to assess whether VF-enabled land sparing is environmentally effective, economically viable, and socially acceptable.

## 6 Conclusion

This study was the first of its kind to consider and quantify the land sparing potential of VF to enable wider environmental benefits. Its aim was to explore the GHG consequences of a transition from field farming to VF when fully integrated with alternative land uses resulting from the land spared by that VF transition.

The findings indicate that VF is not a substitute for field agriculture but a complementary system that can relieve pressure on arable land. By relocating high-yield crops such as lettuce into controlled-environment systems, VF can maintain domestic self-sufficiency while simultaneously sparing land for alternative uses that advance multiple environmental objectives. At the product stage, VF has higher climate change impacts per kg than field lettuce, highlighting that its climate benefits arise primarily from system-level outcomes enabled by land sparing rather than direct production-phase mitigation.

Across alternative land uses, solar combined with VF delivers the greatest reduction in climate change impacts (the only land use when combined with VF which has lower overall impacts than field lettuce production), owing to high energy yields and displacement of fossil electricity, although it incurs relatively high land carbon losses. Agroforestry and forestry show the strongest outcomes for soil carbon and ecosystem services, while peat soils dominate land carbon outcomes and should be restored rather than repurposed to avoid large emissions. No single land use optimises all metrics: solar maximises energy and climate change mitigation, forestry and agroforestry enhance biodiversity and soil carbon, and VF contributes food system resilience and land sparing, albeit at high energy and carbon costs. However, it should be noted that as UK electricity is decarbonised, the relative benefit of installing solar and wind will diminish.

These results underline that outcomes are highly context dependent. Whilst the land-use transitions in this paper are less likely to occur due to the current agricultural land classification system, such work is still important in highlighting potential impacts and their complexities. Inappropriate land uses or poorly managed interventions can negate benefits and generate adverse effects. Multifunctional, adaptive land-use planning that integrates climate, ecological, and societal objectives is therefore essential for sustainable land management in the UK.

## Funding sources

This work was conducted during the first author's PhD study at the Centre for Environment and Sustainability, University of Surrey and the School of Biological Sciences, University of Aberdeen. This work is funded by 10.13039/501100000268BBSRC BB/Z516806/1, 10.13039/501100000268BBSRC PCB4GGR project BB/V011553/1, 10.13039/501100000266EPSRC UKERC-4 10.13039/100015980EP/S029575.

For the purpose of open access, the author has applied a Creative Commons Attribution (CC BY) licence to any Author Accepted Manuscript version arising from this submission.

## CRediT authorship contribution statement

**Michael Gargaro:** Conceptualization, Data curation, Formal analysis, Investigation, Methodology, Project administration, Validation, Visualization, Writing – original draft. **Kaiwen Li:** Data curation, Formal analysis, Methodology, Visualization, Writing – review & editing. **Richard J. Murphy:** Methodology, Project administration, Resources, Software, Supervision, Writing – review & editing. **Astley Hastings:** Funding acquisition, Methodology, Project administration, Resources, Software, Supervision, Writing – review & editing. **Zoe M. Harris:** Funding acquisition, Project administration, Supervision, Writing – review & editing.

## Declaration of competing interest

The authors declare that they have no known competing financial interests or personal relationships that could have appeared to influence the work reported in this paper.

## Data Availability

The authors do not have permission to share data.

## Footnotes

1 An area in the Midlands, UK, which is within 4 hours' drive of 90% of the population (ONS, 2022).

2 Tabular version of results available in Supplementary Section J.

3 Tabular version of results available in Supplementary Section J.

4 Tabular version of results available in Supplementary Section J.

5 We are not suggesting that solar farms should necessarily be placed on prime agricultural land, which likely would be spared for more food production by moving crops from lower graded land to higher grade land, which may liberate further land which is more appropriate for some of these alternative land uses. The analysis is purely indicative of the outcomes that sparing of agricultural land in the UK may render.
