# Supplementary material for: Vertical farming as a land sparing strategy: GHG implications for UK agricultural landscapes
Source: Clean Food Syst. 2026 Jun;3:None. doi: 10.1016/j.clfs.2026.100037 (PMC13256068; doi:10.1016/j.clfs.2026.100037)
Supplement: Multimedia component 1 [file mmc1.docx]

## Supplementary section A - UK lettuce demand calculations

As part of the modelling, there was a need to understand the demand for lettuce in the UK. This is fundamental information for calculating the amount of land which could be spared, as well as what the demand for lettuce is for the UK market. Farming figures below are taken from Defra’s Horticultural statistics (DEFRA, 2025), and population from the UK Office for National Statistics census data for 2021 (ONS, 2025).

Demand of lettuce below is calculated using the 2021 national UK population, divided by the total supply for the UK (excluding exports of lettuce grown within the UK).

Table s1: Calculation of UK lettuce demand and value of imports, domestic production and exports of lettuce

| **Overall Category** | **Area of interest** | **Value** | | **Unit** |
| --- | --- | --- | --- | --- |
| **Field production UK** | Lettuce area UK | | 4001 | ha |
|  | Marketed yield UK (total) | | 88 | thousand tonnes |
| **Protected production UK** | Protected veg area | | 418 | ha |
|  | Protected veg HPM | | 15 | thousand tonnes |
| **UK imports** | Veg imports | | 202 | thousand tonnes |
| **UK exports** | Veg exports | | 4 | thousand tonnes |
| **Total/overall** | Home Production Marketed (HPM) | | 103 | thousand tonnes |
|  | Imports | | 202 | thousand tonnes |
|  | Exports | | 4 | thousand tonnes |
|  | Total Supply for UK market | | 301 | thousand tonnes |
|  | HPM as % of Total Supply | | 34 | % |
| **UK demand** | Population of the UK | | 67,026,000 | people |
|  | Demand of lettuce per person | | 4.5 | kg |

## Supplementary section B - Transport calculations

For both the field and VF scenarios, the same transportation methodology is used. Within VF, the impact for both hyperlocal (HL) and distribution-centre (DC) is calculated separately also.

For all scenarios, the shortest distance possible between either farm and supermarket, farm and DC, or DC and supermarket was calculated. Results for all the individual routes and distances found were outputted, and then used to calculate a single overall average transport distance, which was then used within SimaPro for calculating LCA impacts.

#### Field transport

We used the network analyst tool in ArcMap to calculate the distance between farms and it’s destination of supermarkets. Supermarkets data were obtained from the authors of SWC Maps directly (SWC Maps, 2022). The UK road network dataset from OS open roads was employed to find the shortest routes (Ordnance Survey, 2025). Crop distribution in the UK at present is typically sent from farm to distribution centres (DC), and then from DC to the supermarket. Data regarding the locations of all current DCs in the UK were not available (only available for Lidl, Asda and Tesco): however, other locations for the remaining major supermarkets in the UK (Waitrose, Morrisons, Sainsburys, Aldi) were located using Google (and Google maps). A map of distribution centre locations in the UK was created and used to route lettuce from farms to DCs, and then DCs to supermarkets across the UK (for all field farm produce, delivering to all supermarkets across the UK via one of the DCs).

The transportation distance was calculated by routing all farms to their closest DC, modelling them using a lorry with a 10 T load per journey (450 journeys per year) (using the same vehicle and similar load to previous LCAs by Gargaro et al. (2025). The distances between all DCs in the UK to all supermarkets was then calculated (using the same transport type assumptions).

There are 1,493 different lettuce farm plots, 70 different DC’s and all major supermarkets in the UK (as of January 2022). From every route taken, the average distance between the farm and DC, and then DC to supermarket was calculated, to give the average transportation distance for lettuce in the UK (62.38 km).

#### VF transport

The same approach as the above for field lettuce distribution was used with minor modifications.

Instead of using field‑farm locations, the distance between DCs and supermarkets was used directly as the transport distance. The VF‑DC scenario transport calculations were completed first; the DC‑to‑supermarket transport result from this scenario was then applied to the field‑farming scenario. This approach was taken because the same quantity of lettuce is transported from each DC to each supermarket in both scenarios, with the only difference being that, in the VF‑DC scenario, the VF facilities are co‑located with the DCs.

For the HL scenario however, the transportation was calculated without distribution centres involved. The HL scenario places vertical farms in areas of highest population density across the UK, which typically is where supermarkets are located. Because of this, journey routing was directly from each HL-VF to supermarkets. The same transportation type as in Gargaro et al. (2024) and Gargaro et al. (2025) was used; electric bike courier, with a 150 kg load per journey (1500 journeys per year per facility) (using the same vehicle and similar loads to previous publications).

For both HL and DC scenario, the average distance between VF and supermarket was used, to give the average transportation distance for lettuce in the UK (HL-4.91 km, DC- 26.85 km)

#### Spared Land

Transportation associated with land‑sparing scenarios was fully embedded within the LUNA model. This includes all transport activities required for each land‑use change option, such as the movement of materials, machinery, harvested biomass, and renewable‑energy components, captured consistently within LUNA’s life‑cycle framework (Hastings et al., in preparation).

## Supplementary section C- Soil emissions calculations

The Denitrification-Decomposition (DNDC) model was employed for calculating the soil emissions for every field cultivating lettuce in the UK (DNDC, 2018).


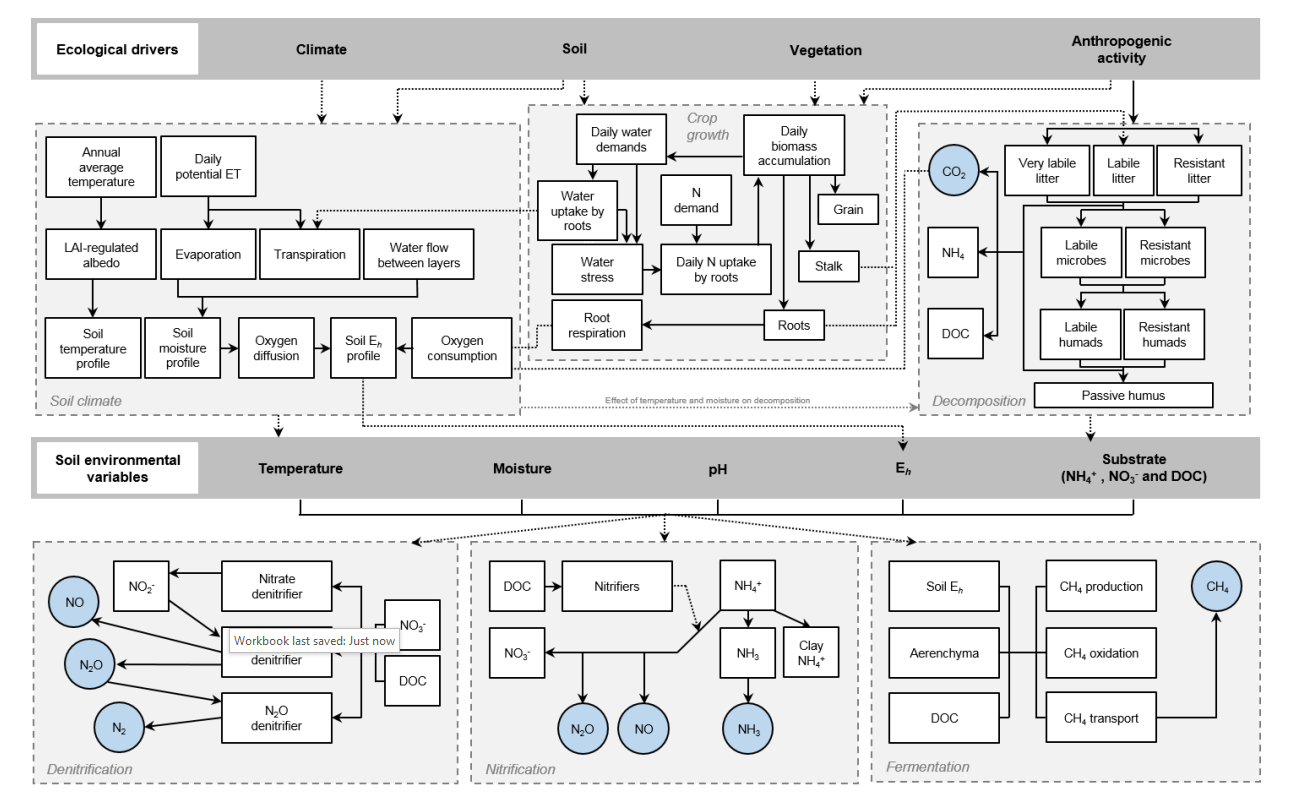


Figure s1: Schematic representation of the DNDC model structure, comprising two components, 1. Ecological drivers (top), and 2. Soil environmental variables (bottom). Taken from Taft et al. (2019) adapted from Li (2000).

Cultivation was split into two overarching categories: mineral soils and peat soils. By area, mineral soils total 80.55%, whereas peat soils cover 19.45%.

The LCA data used were taken from previous work by Gargaro et al. (2025). As the environmental impact using LCA of both a commercial mineral and peat soil-based farm has been previously calculated, the impacts were initially taken as an assumption, assuming the impact on either soil type was the same across the country. Looking deeper into the soil classifications for each plot from HWSD and the peaty soils map of England, a decision was made to calculate the field emissions for all soil types across all different regions of the UK (FAO, 2008; Natural England, 2024). UK climate maps for maximum and minimum temperature, and precipitation were loaded into ArcMap (Met Office, 2024), which showed the variation of climate across the UK, as well as the variation in soil types used for cultivating lettuce from the soil map layers.

To calculate the soil emission from the land, data were extracted for soil types used for lettuce cultivation in the UK from the previously extracted areas (the 1493). HWSD reports them as individual soil types (Eutric Fluvisol, Calcic Fluvisol etc.), but we grouped them into their overall soil type (in this case it would be Fluvisol). There were 7 different soil types overall:

- Histosol- 22% land area
- Arenosol- 0.5% land area
- Cambisol- 8.5% land area
- Fluvisol- 32% land area
- Gleysol- 7% land area
- Luvisol- 27% land area
- Podzol- 3% land area

Each individual soil type was run with its regional climate data (dependant on region and data availability, ranging from 2014-2022 data). This was not run for every single iteration of a soil type in each region, but instead took the most common/average soil parameters for each soil in each region and ran those through DNDC with the right regional climate data. The majority of each overall soil type in each region had almost identical soil characteristics, hence the most common/average value was used as opposed to running each individual field plot.

With these results, there is now a more granular picture of the impacts across the UK for lettuce cultivation per region. However, there are still assumptions with this method which are outlined below:

- Though soil and climate maps can give many of the details about the conditions in which the crop is being grown, it does not give information about how cultivation happens in those regions (i.e. additional water inputs, fertiliser and manure applications (and frequency), or the yields per area). This information was based on previously acquired data from commercial lettuce farms (one mineral soil and one peat), with the assumption that the inputs remain the same for all types of mineral and peat soils, except for their soil parameters and climatic conditions (Gargaro et al., 2025). The data and methods for calculating impacts from soils using DNDC, as well as the other inputs of fertilisers, water, manure, tractor use, yields, can be found in Gargaro et al. (2025).
- Assumption that all mineral soils use the same quantity of inputs, and yield the same amount of crop across the UK, regardless of soil type, climate conditions or region. The same was applied for peat soils.
- The different soil emissions from the various soils in all regions were added into the LCA. This was done based on the proportion of each soil type as a percentage of total land area for cultivating lettuce in the UK and as its percentage of either mineral or peat soil. These were totalled for mineral and peat individually, and added into the overall LCA result. This means that the soil‑emissions values represent a weighted national average rather than emissions from any single soil type. Mineral and peat soils contribute to the final figures according to their proportion of UK field‑grown lettuce production. Since mineral soils represent 80.5% of the land used for lettuce and peat soils 19.5%, the aggregated soil‑emissions result is composed of 80.5% mineral‑soil emissions and 19.5% peat‑soil emissions.

Table s2: Crop input data used for DNDC soil emission modelling. Crop input data was taken from Gargaro et al. (2025); UK1M is the mineral soil lettuce farm, and UK2M is the peat soil lettuce farm data. Soil and climate data inputs were recalculated for this study.

| Input variable | | Units | Field | |
| --- | --- | --- | --- | --- |
|  | |  | Mineral soil lettuce farm  (UK 1 M) | Peat soil lettuce farm  (UK 2 M) |
| Crop type | |  | (34, Lettuce) | (34, Lettuce) |
| Perennial crop? | | Yes/No | (No) | (No) |
| Cover crop? | | Yes/No | (No) | (No) |
| Fraction of leaves and stems left in the field after harvest | | Fraction (0-1) | (1) | (1) |
| Planting and harvesting dates | |  | 13/05-04/07 | 23/05-15/07 |
| Max biomass production | Grain | kg C ha^-1^ yr^-1^ | 288 | 660 |
|  | Leaf |  | (36) | (82.5) |
|  | Stem |  | (36) | (82.5) |
|  | Root |  | (90) | (206.25) |
| Biomass fraction | Grain | Fraction 0-1 | (0.64) | (0.64) |
|  | Leaf |  | (0.08) | (0.08) |
|  | Stem |  | (0.08) | (0.08) |
|  | Root |  | (0.2) | (0.2) |
| Biomass C:N ratio | Grain | Ratio | (11.5) | (11.5) |
|  | Leaf |  | (20) | (20) |
|  | Stem |  | (20) | (20) |
|  | Root |  | (30) | (30) |
| Annual N demand | | kg N ha^-1^ yr^-1^ | (31.64) | (72.52) |
| TDD for maturity | |  | 496.95 | 567 |
| Water demand | | g H_2_O g DM^-1^ | (800) | (800) |

## Supplementary section D - Spanish cultivation scenario

Previous work by the authors have calculated the LCA impacts of delivery of Spanish cultivated lettuce for the UK market, available in Gargaro et al. (2025), underpinned by data from Milà Canals et al. (2009). LCI data for Spanish scenarios is available in Table s5 in Supplementary Section H.

In 2021, Spanish lettuce production was 1,063,775 tonnes (Gobierno de Espana, 2021). 811,704 tonnes is exported annually (FEPEX, 2022). UK imports 201.7 thousand tonnes (of which 91.95% is from Spain) (DEFRA, 2025; HortoInfo, 2024). The land area for cultivating in Spain is 34,150 hectares (Gobierno de Espana, 2021). According to Spanish data, the UK in 2020 imported 128,635 thousand tonnes of lettuce (FEPEX, 2022). Though mixing years (as no value for export to the UK from Spain in 2021 from official Spanish statistics exists, as the UK is no longer in the EU, the value is hidden within a generic non-EU export), the land area used in Spain for cultivating lettuce is 4,129.52 ha.

UK population in 2021 was: 67,026,000 (ONS, 2025). The lettuce per person (based on UK consumption of 300.8 thousand tonnes) was 4.49 kg, which was rounded up to 4.5 kg for simplicity in analysis (minor overestimate in the amount of lettuce per person). The land spared areas for outside the UK are also presented for transparency, and to show that not all land will be spared within the UK. The UK imports 91.95% of its lettuce from Spain, over an area in Spain of 4,130 ha. If we assume all other areas which export to the UK yield relatively similar amounts of lettuce per area, then the land spared outside the UK in total would be 4,490 ha.

## Supplementary section E- Description of VF scenarios

### HL

Within the HL scenario, VF facilities were sited in locations optimised for UK population density to minimise transportation distances. ArcMap was used to look at the population density of the country; the population density of each local authority (LA) itself and its density in relation to the rest of the country (Carnell & Tomlinson, 2025; ONS, 2024a, 2024b). Based on this, a facility which could feed 50,000 people was selected. The average size of a local authority was approximately 175,000 people. These VF facilities are spread within each LA, and rounded up to ensure there is sufficient lettuce production for each LA (i.e, in Enfield borough (North London), the population is c.330,000 people, so we placed 7 facilities within Enfield borough (6 plus the one to fulfil the remaining 30,000 people, plus 20,000). With this approach, there inherently will be overproduction of lettuce in the UK.

According to the UK population in 2021, 1,341 facilities are needed to feed the population exactly, but with this approach where VF proximity to the population is prioritised, this results in a total of 1,515 facilities, resulting in an overproduction of 174 facilities (which could feed an extra 8,700,000 people). However, this analysis is still assuming that each LA will only produce enough lettuce for its own population (i.e., for Enfield, there are 7 facilities, but each facility will produce lettuce for 47,142 people). Additionally, this established overproduction could work in favour of VF and future proofing facilities. In the UK, the population is predicted to grow to 76.6 million by 2047 (which is ~8 million more than present) (ONS, 2025).

### DC

Within the DC scenario, the aim was to mirror current business-as-usual distribution of crops across the UK. Instead of growing crops near population centres, VF’s were sited similarly to how DCs are in the UK at present, mostly inside the golden triangle of logistics in the UK (being within a 4 hour drive of 90% of the UK population) (ONS, 2022), as well as depots in other major regions or more remote regions (Borderlands, Central belt, Bristol, South-West), plus some additional ones derived from population density (North-West (Liverpool, Leeds, Manchester), South coast (Brighton), North Scotland (Aberdeen)). This resulted in 70 facilities for the UK, with each facility feeding 1mn people. This is roughly representative of the amount of DC’s for food for the major supermarkets in the UK (roughly 15-20 each). Tesco for example has 20 DC’s, with 29% market share in the UK (Bedford, 2025; NX, 2025)

### Assumptions (both HL and DC)

- Assumed that anywhere in the local authority was acceptable to put a VF facility. No consideration was given to the availability of land, the price of land etc.
- Assuming lettuce isn’t bought anywhere except major supermarkets (small retailers are not included, but convenience store versions of major supermarkets, i.e. Tesco express’, are).
- Assumed there is a consistent demand rate throughout the year for lettuce, as well as equal demand for lettuce throughout the entire UK geographically.
- Assuming a 1:1 economy of scale for VF systems. I.e., a 50k people facility and a 1mn people facility have the same impact per kg of crop produced. Partner VF companies indicated there are no economy of scale benefits of having a much larger facility in terms of environmental impact. In terms of construction costs however, the unit construction cost of VFs can decline by as much as 55% when the scale of VFs is increased 100 times (Zhuang et al., 2022).

## Supplementary Section F- System Boundaries

Figures s2-s4 represent the more detailed system boundaries for the individual components of the modelling completed in this study.

For more detailed information on each scenario, please refer to Gargaro et al. (2024) for VF scenarios, Gargaro et al. (2025) for field farming scenarios, and Hastings et al. (in preparation) for the land sparing scenarios using LUNA.


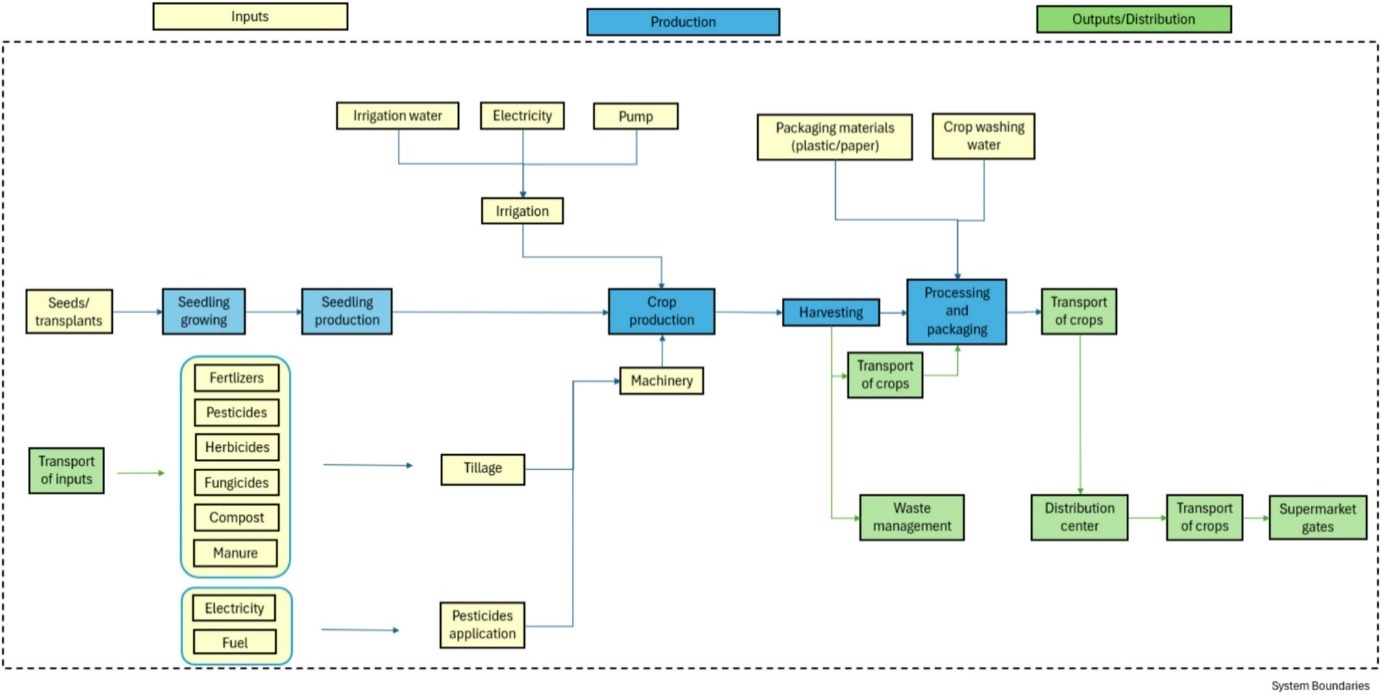


Figure s2: System boundaries for field farmed lettuce scenarios in this study. Taken from Gargaro et al. (2025).


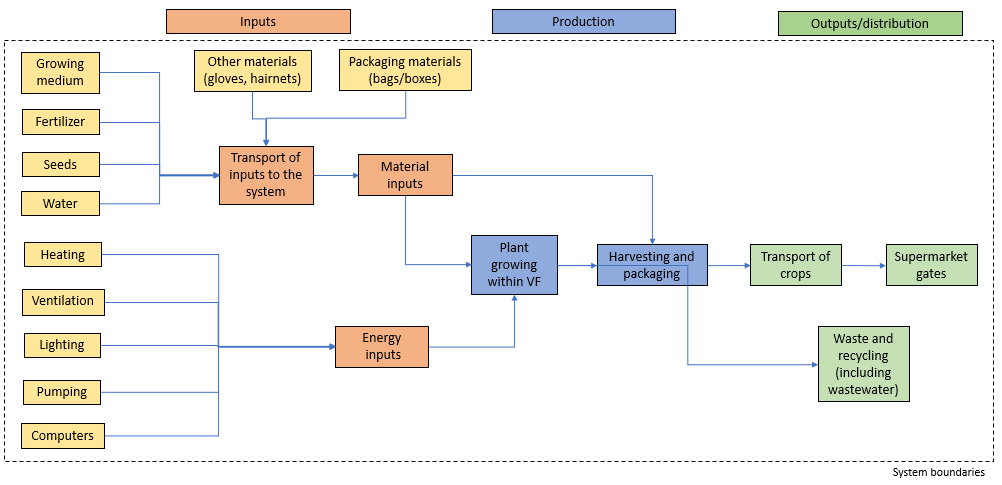


Figure s3: System boundary for the cultivation of lettuce within a vertical farm in the UK, from cradle-to-supermarket. Taken from Gargaro et al. (2024) and adapted.


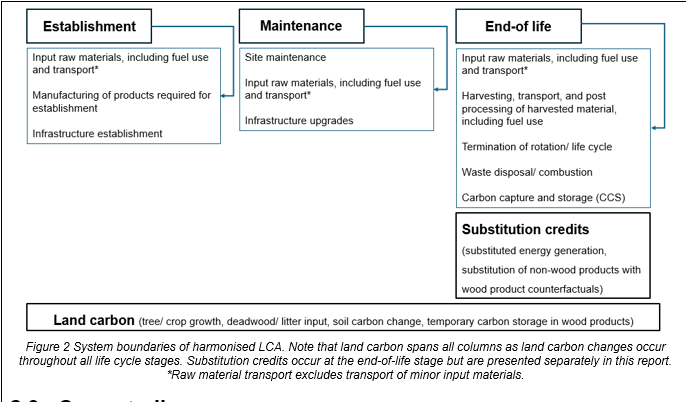


Figure s4: System boundaries of harmonised LCA for the LUNA model. Taken from Hastings et al. (in preparation).

## Supplementary section G - Soil emissions per soil type and region

Table s3: Soil emissions calculated per region and soil type in the UK. Naming for each region and soil type is presented as "W*-A**" (*indicates the regions, ** indicates the soil type). REGIONS: W-West, S/SW- South and South-West, SE- South-East, E-East, NE- North-East, NW- North-West. SOIL TYPE: A- Arenosol, C- Cambisol, F- Fluvisol, G- Glesysol, L- Luvisol, H- Histosol, P- Podzol.

| **Soil type and region/ GHG** | **CO_2_** | **N_2_0** | **CH_4_** |
| --- | --- | --- | --- |
| ***Unit*** | ***kg CO_2_ / kg crop*** | ***kg N / kg crop*** | ***kg C / kg crop*** |
| W-A | -0.00658 | 0.00001 | 0.00000 |
| W-C | 0.00988 | 0.00003 | 0.00000 |
| W-F | 0.00196 | 0.00003 | 0.00000 |
| W-G | -0.00063 | 0.00003 | 0.00000 |
| W-L | 0.00067 | 0.00003 | 0.00000 |
| S/SW-H | 0.27963 | 0.00015 | -0.00007 |
| S/SW-A | -0.00646 | 0.00001 | 0.00000 |
| S/SW-C | 0.01013 | 0.00004 | 0.00000 |
| S/SW-F | -0.00033 | 0.00004 | 0.00000 |
| S/SW-G | -0.00033 | 0.00003 | 0.00000 |
| S/SW-L | 0.00067 | 0.00004 | 0.00000 |
| SE-C | -0.00017 | 0.00018 | 0.00000 |
| SE-F | -0.00058 | 0.00013 | 0.00000 |
| SE-G | 0.00021 | 0.00017 | 0.00000 |
| SE-L | 0.00138 | 0.00019 | 0.00000 |
| E-H | 0.23944 | 0.00009 | -0.00005 |
| E-A | -0.00783 | 0.00001 | 0.00000 |
| E-C | -0.00313 | 0.00001 | 0.00000 |
| E-F | -0.00729 | 0.00000 | 0.00000 |
| E-G | 0.00783 | 0.00005 | 0.00000 |
| E-L | 0.00179 | 0.00003 | 0.00000 |
| NE-H | 0.24137 | 0.00004 | -0.00007 |
| NE-C | -0.00108 | 0.00003 | 0.00000 |
| NE-F | -0.00617 | 0.00001 | 0.00000 |
| NE-G | 0.00558 | 0.00003 | 0.00000 |
| NE-L | 0.00450 | 0.00004 | 0.00000 |
| NW-H | 0.27095 | 0.00010 | -0.00005 |
| NW-C | 0.00000 | 0.00002 | 0.00000 |
| NW-F | -0.00354 | 0.00001 | 0.00000 |
| NW-L | 0.00492 | 0.00004 | 0.00000 |
| NW-P | 0.02246 | 0.00001 | -0.00004 |

Figure s5: Soil emissions per region and soil type in the UK. Naming for each region and soil type is presented as "W*-A**" (*indicates the regions, ** indicates the soil type). REGIONS: W-West, S/SW- South and South-West, SE- South-East, E-East, NE- North-East.

## Supplementary section H - Life Cycle Inventory (LCI)

For VF scenarios, Table s4 has LCI data for the VF scenarios.

For field farming scenarios, Table s5 has the LCI data. Mineral soil LCI data is for UK1, peat soil LCI data is for UK2. Spanish soil LCI data is an average of Sp 1-4.

This life-cycle inventory data is taken from and available in Gargaro et al. (2024) and Gargaro et al. (2025). The former has detailed LCI for VF scenarios, the latter for field farming scenarios (and also VF scenarios). Please refer to both papers’ main manuscript and supplementary materials for further details.

Additionally, as stated in this paper’s main manuscript, the only changes from previous LCIs is the transportation of lettuce from farm to supermarket, and soil emissions.

Table s4: LCI data for the VF (inputs and outputs normalised to the Functional Unit). Taken from Gargaro et al. (2025).

| Stage | Flow | Process/activity | Value | Units |
| --- | --- | --- | --- | --- |
| Transport of inputs to the farm | Input | Seed delivery | 0.077 | kg km^-1^ |
|  |  | Chemical and nutrient delivery | 15.25 | kg km^-1^ |
|  |  | Grow media and plug delivery | 28.22 | kg km^-1^ |
| Cultivation stage | Input | Seeds | 0.0005 | kg |
|  |  | Calcium Nitrate | 0.008 | kg |
|  |  | Magnesium Sulphate | 0.008 | kg |
|  |  | Solufeed F | 0.020 | kg |
|  |  | TEC | 0.00066 | kg |
|  |  | Nitric Acid (25%) | 0.034 | kg |
|  |  | Hydrogen Peroxide (50%) | 0.045 | kg |
|  |  | Grid electricity (all stages) | 17.25 | kWh |
|  |  | Water (all stages) | 0.028 | m^3^ |
|  |  | Jute fibre plugs | 0.063 | kg |
|  |  | Bin liners | 0.043 | kg |
| Harvesting and packaging | Input | Reuseable plastic crate | 0.003 | kg |
|  |  | Labels | 0.00068 | kg |
| Cleaning and disposables | Input | Surface disinfectant | 0.0014 | kg |
|  |  | Surface detergent | 0.0014 | kg |
|  |  | Bactericidal washing liquid | 0.0021 | kg |
|  |  | Latex gloves | 0.0010 | kg |
| Outputs | Output | Lettuce to customer | 1.00 | kg |
|  |  | Waste Lettuce | 0.075 | kg |
|  |  | General waste | 1.35 | kg |
|  |  | Recycling waste | 0.035 | kg |
|  |  | Wastewater | 0.035 | m^3^ |
|  |  | Nutrient waste (all nutrients by element) | 0.030 | mg l^-1^ |
| Transport of outputs from the system | Output | Delivery to customer | 0.15 | kg km^-1^ |
|  |  | Waste collection | 32.03 | kg km^-1^ |

Table s5: LCI data for the field farming scenarios (inputs and outputs normalised to the Functional Unit). Taken from Gargaro et al. (2025).*** UK soil emissions for this study have been recalculated (except for spanish production, which remains the same as previously published).

| Stage | Flow | Process/activity | Units | UK 1 | UK 2 | Sp 1 | Sp 2 | Sp 3 | Sp 4 |
| --- | --- | --- | --- | --- | --- | --- | --- | --- | --- |
| Transport of inputs to the farm | Input | Seed/transplant delivery | kg km^-1^ | 53.94 | 0.013 | 0.000280 | 0.000280 | 0.000280 | 0.000280 |
|  |  | Chemical and nutrient delivery | kg km^-1^ | 2.65 | 1.82 | 7 | 7 | 76.51 | 92 |
|  |  | Diesel delivery | kg km^-1^ | 4.06 | 1.56 | 0.53 | 0.53 | 0.33 | 0.36 |
|  |  | Other inputs (packaging and disposables) | kg km^-1^ | 29.23 | 0.42 | - | - | - | - |
| Cultivation stage | Input | Seeds/transplants | kg | 0.000005 | 0.0000022 | 0.0000035 | 0.0000035 | 0.0000035 | 0.0000035 |
|  |  | Seeded blocks | kg | 0.000044 | 0.000044 | 0.00033 | 0.00033 | 0.00022 | 0.00027 |
|  |  | Fertilisers (all) | kg | 0.074 | 0.035 | 0.026 | 0.026 | 0.0056 | 0.0053 |
|  |  | Pesticides (all) | kg | 0.00023 | 0.000072 | 0.0012 | 0.0012 | 0.0023 | 0.002 |
|  |  | Herbicides (all) | kg | 0.00022 | 0.0002 | 0.001 | 0.001 | 0.001 | 0.001 |
|  |  | Fungicides (all) | kg | 0.00016 | 0.00008 | 0.0006 | 0.0006 | 0.0006 | 0.0006 |
|  |  | Compost/manure | kg | 0.053 | - | 0.15 | 0.15 | 1.81 | 2.17 |
|  |  | Crop fleece | kg | 0.011 | 0.00051 | - | - | - | - |
|  |  | Diesel consumption (all) | kg | 0.04 | 0.021 | 0.006 | 0.006 | 0.0037 | 0.004 |
|  |  | Grid electricity (all stages) | kWh | 0.12 | 0.075 | 0.08 | 0.12 | 0.085 | 0.17 |
|  |  | Water (all stages) | m^3^ | 0.023 | 0.00021 | 0.042 | 0.074 | 0.023 | 0.09 |
| Harvesting, packaging and disposables | Input | Reuseable plastic crate | kg | - | * | - | - | - | - |
|  |  | Polypropylene packaging | kg | 0.0033 | - | - | - | - | - |
|  |  | Wholehead film | kg | 0.0049 | - | - | - | - | - |
|  |  | Labels | kg | 0.0005 | - | - | - | - | - |
|  |  | Cardboard boxes | kg | 0.02 | - | - | - | - | - |
|  |  | Latex gloves | kg | 0.00015 | 0.0000067 | - | - | - | - |
| Outputs | Output | Lettuce to customer | kg | 1 | 1 | 1 | 1 | 1 | 1 |
|  |  | Waste Lettuce | kg | 0.074 | 0.055 | 0.2 | 0.2 | 0.2 | 0.2 |
|  |  | General waste | kg | 0.0028 | 0.00062 | 0.2 | 0.2 | 0.2 | 0.2 |
|  |  | Recycling waste | kg | 0.0036 | 0.0066 | 0.019 | 0.019 | 0.019 | 0.019 |
|  |  | Green waste (to AD or composted) | kg | 0.0014 | - | - | - | - | - |
|  |  | Wastewater | kg | - | - | - | - | - | - |
|  |  | Soil emissions- N_2_O*** | kg | *** | *** | -0.018 | -0.016 | -0.3 | -0.36 |
|  |  | Soil emissions- CO_2_*** | kg | *** | *** | 0.000033 | 0.000047 | 0.00024 | 0.00082 |
|  |  | Soil emissions- CH_4_*** | kg | *** | *** | 0.00028 | 0.000028 | 0.000028 | 0.000028 |
| Transport of outputs from the system | Output | Delivery to supermarket (via the DC) | kg km^-1^ | 169.28 | 170 | 2600 | 2600 | 2600 | 2600 |
|  |  | Waste collection | kg km^-1^ | 0.11 | 0.19 | 5.08 | 5.08 | 5.08 | 5.08 |

## Supplementary section I- Description of LUNA model.


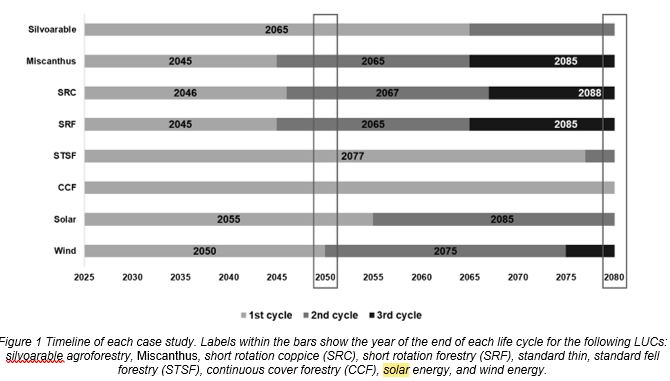


Figure s6: Temporal cycle between establishment and end-of-life for each land use change scenario in LUNA. Labels within the bars show the year of the end of each lifecycle for the following LUCs: silvoarable agroforestry, Miscanthus, SRC willow, SRF, STSF, CCF, solar energy, wind energy (Hastings et al., in preparation)

LUNA assesses the impacts of various land use change (LUC) scenarios across Great Britain, enabling comparisons of soil and vegetation carbon dynamics and quantifies the carbon intensity of each transition (Hastings et al., in preparation). The model is underpinned by LCA methodology and calculates total GHG emissions resulting from converting notional 1-hectare areas from their 2025 known baseline use to alternative LUC scenarios (Hastings et al., in preparation). Results are expressed as cumulative GHG emissions per hectare through 2050, aligning with the UK’s Net Zero target year. Each LUC scenario follows its own distinct temporal cycle between establishment and end-of-life (Hastings et al., in preparation).

Results generated by the LUNA model cover the period 2025–2050, representing a 25-year timeframe (Hastings et al., in preparation). These outcomes are not linear year-on-year; for example, the benefits of woodland planting accumulate gradually rather than occurring immediately (Hastings et al., in preparation). For this reason, most results (including those for field-grown and vertically farmed lettuce) are reported over the full 25-year period, with annual breakdowns provided where relevant. For lettuce systems, 25-year impacts are calculated by multiplying the one-year LCA results by 25.

## Supplementary Section J - Land sparing results

Table s6: LCA impacts (in kg CO_2_ eq) for all alternative land use scenarios; output for period 2025-2050 (25 years) and average of 1 year.

| **Land use type** | **Total 25 years** | **Unit** | **Total 1 year** | **Unit** |
| --- | --- | --- | --- | --- |
| *Miscanthus* unabated (Climate Change) | 7.46E+08 | kg CO_2_ eq | 2.98E+07 | kg CO_2_ eq |
| *Miscanthus* BECCS | 9.74E+07 | kg CO_2_ eq | 3.90E+06 | kg CO_2_ eq |
| SRC Willow unabated | 9.35E+08 | kg CO_2_ eq | 3.74E+07 | kg CO_2_ eq |
| SRC Willow BECCS | 2.31E+08 | kg CO_2_ eq | 9.24E+06 | kg CO_2_ eq |
| 2MW wind turbine | 6.20E+08 | kg CO_2_ eq | 2.48E+07 | kg CO_2_ eq |
| 4MW wind turbine | 3.28E+08 | kg CO_2_ eq | 1.31E+07 | kg CO_2_ eq |
| 6MW wind turbine | 1.10E+09 | kg CO_2_ eq | 4.40E+07 | kg CO_2_ eq |
| Solar | -4.05E+09 | kg CO_2_ eq | -1.62E+08 | kg CO_2_ eq |
| Agroforestry unabated | 5.44E+07 | kg CO_2_ eq | 2.18E+06 | kg CO_2_ eq |
| Agroforestry BECCS | 5.44E+07 | kg CO_2_ eq | 2.18E+06 | kg CO_2_ eq |
| Broadleaved CCF unabated | 7.28E+08 | kg CO_2_ eq | 2.91E+07 | kg CO_2_ eq |
| Broadleaved CCF BECCS | 7.14E+08 | kg CO_2_ eq | 2.86E+07 | kg CO_2_ eq |
| Broadleaved STSF unabated | 7.28E+08 | kg CO_2_ eq | 2.91E+07 | kg CO_2_ eq |
| Broadleaved STSF BECCS | 7.14E+08 | kg CO_2_ eq | 2.86E+07 | kg CO_2_ eq |
| Conifer CCF unabated | 8.97E+07 | kg CO_2_ eq | 3.59E+06 | kg CO_2_ eq |
| Conifer CCF BECCS | 3.63E+07 | kg CO_2_ eq | 1.45E+06 | kg CO_2_ eq |
| Conifer STSF unabated | 8.97E+07 | kg CO_2_ eq | 3.59E+06 | kg CO_2_ eq |
| Conifer STSF BECCS | 3.63E+07 | kg CO_2_ eq | 1.45E+06 | kg CO_2_ eq |
| SRF BECCS | 2.97E+08 | kg CO_2_ eq | 1.19E+07 | kg CO_2_ eq |
| SRF unabated | 3.73E+08 | kg CO_2_ eq | 1.49E+07 | kg CO_2_ eq |

Table s7: Land carbon impacts (in kg C) for all alternative land use scenarios; output for period 2025-2050 (25 years) and average of 1 year.

| **Land use type** | **Unit** | **Land carbon 25 Y** | **Land carbon 1 Y** |
| --- | --- | --- | --- |
| *Miscanthus* | kg C | 2.04E+08 | 8.14E+06 |
| SRC Willow | kg C | 2.50E+08 | 9.99E+06 |
| 2MW wind turbine | kg C | 9.51E+07 | 3.80E+06 |
| 4MW wind turbine | kg C | 1.87E+08 | 7.46E+06 |
| 6MW wind turbine | kg C | 3.12E+08 | 1.25E+07 |
| Solar | kg C | 2.65E+08 | 1.06E+07 |
| Agroforestry | kg C | 1.50E+07 | 6.01E+05 |
| Broadleaved CCF | kg C | 1.96E+08 | 7.86E+06 |
| Broadleaved STSF | kg C | 1.96E+08 | 7.86E+06 |
| Conifer CCF | kg C | 3.31E+07 | 1.33E+06 |
| Conifer STSF | kg C | 3.31E+07 | 1.33E+06 |
| SRF | kg C | 5.37E+07 | 2.15E+06 |

Table s8: Energy production (in KWh) for all alternative land use scenarios; output for period 2025-2050 (25 years) and average of 1 year.

| **Land use type** | **Unit** | **Energy Production 25 Y** | **Energy production 1 Y** |
| --- | --- | --- | --- |
| *Miscanthus* unabated (Climate Change) | kWh | 5.22E+08 | 2.09E+07 |
| *Miscanthus* BECCS | kWh | 3.85E+08 | 1.54E+07 |
| SRC Willow unabated | kWh | 5.18E+08 | 2.07E+07 |
| SRC Willow BECCS | kWh | 4.00E+08 | 1.60E+07 |
| 2MW wind turbine | kWh | 1.21E+10 | 4.84E+08 |
| 4MW wind turbine | kWh | 1.52E+10 | 6.08E+08 |
| 6MW wind turbine | kWh | 1.90E+10 | 7.61E+08 |
| Solar | kWh | 6.23E+10 | 2.49E+09 |
| Agroforestry unabated | kWh | 0.00E+00 | 0.00E+00 |
| Agroforestry BECCS | kWh | 0.00E+00 | 0.00E+00 |
| Broadleaved CCF unabated | kWh | 1.05E+07 | 4.22E+05 |
| Broadleaved CCF BECCS | kWh | 8.01E+06 | 3.21E+05 |
| Broadleaved STSF unabated | kWh | 1.05E+07 | 4.22E+05 |
| Broadleaved STSF BECCS | kWh | 8.01E+06 | 3.21E+05 |
| Conifer CCF unabated | kWh | 4.04E+07 | 1.62E+06 |
| Conifer CCF BECCS | kWh | 3.07E+07 | 1.23E+06 |
| Conifer STSF unabated | kWh | 4.04E+07 | 1.62E+06 |
| Conifer STSF BECCS | kWh | 3.07E+07 | 1.23E+06 |
| SRF BECCS | kWh | 2.07E+07 | 8.28E+05 |
| SRF unabated | kWh | 2.72E+07 | 1.09E+06 |
| Energy demand of VF for all 300mn | kWh | 1.30E+11 | 5.19E+09 |
| Energy demand of VF if only growing UK spared land crop (88mn) | kWh | 3.80E+10 | 1.52E+09 |

## Supplementary section K- Land carbon results without peat soils

Table s9: Land carbon impacts (in kg C) for all alternative land use scenarios (with peat soils removed); output for period 2025-2050 (25 years) and average of 1 year.

| **Land use type** | **Unit** | **Land Carbon 25 Y** |
| --- | --- | --- |
| 6MW wind turbine | kg C | 7.92E+07 |
| Solar | kg C | 5.16E+07 |
| SRC Willow | kg C | 1.38E+07 |
| Miscanthus | kg C | -3.02E+07 |
| Broadleaved CCF | kg C | -4.08E+07 |
| Broadleaved STSF | kg C | -4.08E+07 |
| 4MW wind turbine | kg C | 4.74E+07 |
| 2MW wind turbine | kg C | 2.42E+07 |
| SRF | kg C | 9.97E+06 |
| Conifer CCF | kg C | -1.95E+08 |
| Conifer STSF | kg C | -1.95E+08 |
| Agroforestry | kg C | -2.69E+06 |


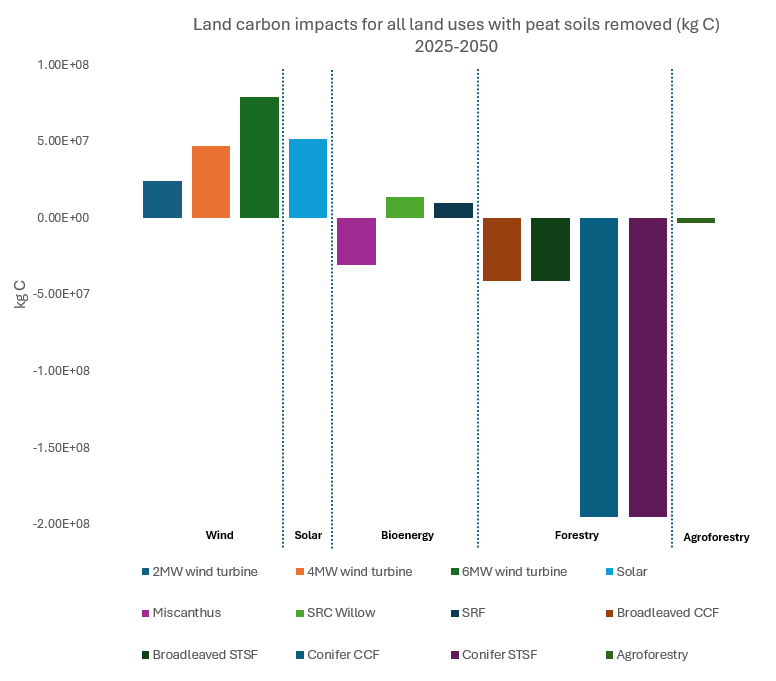


Figure s7: Land carbon impacts (in kg C) for all alternative land use scenarios evaluated on land spared from lettuce production in the UK with peat soils removed (land carbon impacts are the same for unabated and BECCS scenarios); output for period 2025-2050 (25 years). Lower land carbon values correspond to greater sequestration and therefore stronger climate benefits, while higher values indicate net carbon loss.

## Supplementary section L- Global average emission factors for restored peatlands

Secondary literature data were used to find a global average for net GHG emissions of rewetted and restored peatlands per area (m^2^) per year. Data from 31 studies (listed below) was collected and analysed, and categorised by peatland type (bog or fen). Data extracted from papers were methane emissions, nitrous oxide emissions and carbon dioxide emissions. Calculations for impact were done for both bog and fen separately.

The Interquartile Range (IQR) approach was combined with the 95% Confidence Interval (CI) method to ensure that the statistical range accurately reflects the central tendency of the collected literature data. The IQR method was first applied to remove extreme outliers (Equation 1), which can significantly distort the mean and inflate the standard deviation. Such distortions would otherwise lead to an unrealistically wide 95% CI and reduce its interpretive value. By filtering out anomalous values through the IQR approach, the dataset ($X_{f}$) becomes more stable and approximately normal, allowing the 95% CI (Equation 2) to reflect the true variability of the typical observations rather than being driven by a few extreme cases. This combination thus enhances both the robustness and reliability of the statistical analysis.

Equation 1

$$X_{f}=\left\{ x_{i}\in X \mid Q_{1}-1.5*IQR\leq xi\leq Q_{3}+1.5*IQR \right\}, IQR=Q_{3}-Q_{1}$$

Where: $X$ is the dataset; $X_{f}$ is the filtered dataset by IQR method; $Q_{1}$ and $Q_{3}$ were defined as the 25th and 75th percentiles of the ordered data and were estimated using linear interpolation; $IQR$ is the interquartile range, defined as the difference between the 75th and 25th percentiles of the data; $xi$ represents an individual data point (observation) from the original dataset ($X$) with $i=1,2,3\ldots n$.

Equation 2

$$CI95\%=\bar{x_{f}}\pm t_{0.975, n_{f}-1}*\frac{s_{f}}{\sqrt{n_{f}}}$$

Where: $CI95\%$ is a 95% confidence interval of the filtered dataset ($X_{f}$); $\bar{x_{f}}$ is the mean of filtered dataset ($X_{f}$); $n_{f}$ represents the sample size of filtered dataset ($X_{f}$);

$s_{f}$ is the standard deviation of filtered dataset ($X_{f}$).

Having calculated upper and lower bounds for fens and bogs, the total upper and lower bounds of both combined were calculated, and then a total mean (Table s10). This total mean value (88.6 g CO_2_ eq m^-2^ yr ^-1^) was then used to calculate the overall emissions from restored peatland on the peat soil area used for lettuce in the UK for this study, resulting in peatland restoration emissions of 1.45 × 10⁵ kg CO₂ eq.

Table s10: Global average emission factors for restored peatlands (g CO_2_e m^-2^ yr^-1^).

| Peatland | CH_4_ Flux | CO_2_ Flux | N_2_O Flux | CH_4_ GWP₁₀₀ | N_2_O GWP₁₀₀ | CH_4_ emissions | CO_2_ emissions | N_2_O emissions | 95%CI Net GHG emission factor |
| --- | --- | --- | --- | --- | --- | --- | --- | --- | --- |
|  | g C m⁻² yr⁻¹ | | g m^−2^ yr^−1^ | n/a | n/a | g CO_2_e m^-2^ yr^-1^ | | | |
| Bog_Lower bound | 4.3 | -48.5 | 0.041 | 29.8 | 273 | 172.3 | -177.9 | 11.2 | 5.7 |
| Bog_Upper bound | 8.5 | 5.0 | 0.068 | 29.8 | 273 | 339.0 | 18.2 | 18.5 | 375.7 |
| Fen_Lower bound | 6.2 | -158.3 | 0.031 | 29.8 | 273 | 246.5 | -580.5 | 8.5 | -325.5 |
| Fen_Upper bound | 10.4 | -58.9 | 0.426 | 29.8 | 273 | 411.5 | -216.1 | 116.3 | 311.8 |
| Total_Lower bound | 6.5 | -102.1 | 0.043 | 29.8 | 273 | 256.5 | -374.2 | 11.7 | -106.0 |
| Total_Upper bound | 9.4 | -46.8 | 0.301 | 29.8 | 273 | 372.7 | -171.7 | 82.2 | 283.2 |
| Bog mean | 6.4 | -21.8 | 0.054 | 29.8 | 273 | 255.6 | -79.8 | 14.9 | 190.7 |
| Fen mean | 8.3 | -108.6 | 0.229 | 29.8 | 273 | 329.0 | -398.3 | 62.4 | -6.8 |
| Total mean | 7.9 | -74.5 | 0.172 | 29.8 | 273 | 314.6 | -273.0 | 46.9 | 88.6 |

List of papers included

- Cooper, M. D. A., Evans, C. D., Zielinski, P., Levy, P. E., Gray, A., Peacock, M., ··· Freeman, C. (2014). Infilled ditches are hotspots of landscape methane flux following peatland re-wetting. Ecosystems, 17, 1227–1241.
- Komulainen, V. M., Nykänen, H., Martikainen, P. J., & Laine, J. (1998). Short-term effect of restoration on vegetation change and methane emissions from peatlands drained for forestry in southern Finland. Canadian Journal of Forest Research, 28(3), 402–411.
- Strack, M., & Zuback, Y. C. A. (2013). Annual carbon balance of a peatland 10 yr following restoration. Biogeosciences, 10, 2885–2896.
- Urbanova, Z., Picek, T., & Tuittila, E. S. (2013). Sensitivity of carbon gas fluxes to weather variability on pristine, drained and rewetted temperate bogs. Mires and Peat, 11, 1–14.
- Vanselow-Algan, M., Schmidt, S. R., Greven, M., Fiencke, C., Kutzbach, L., & Pfeiffer, E. M. (2015). High methane emissions dominated annual greenhouse gas balances 30 years after bog rewetting. Biogeosciences, 12, 4361–4371.
- Waddington, J., & Day, S. (2007). Methane emissions from a peatland following restoration. Journal of Geophysical Research-Biogeosciences, 112(G3), pp. 11.
- Wilson, D., Farrell, C. A., Muller, C., Hepp, S., & Renou-Wilson, F. (2013). Rewetted industrial cutaway peatlands in western Ireland: A prime location for climate change mitigation? Mires and Peat, 11(1), 1–22.
- Yli-Petäys, M., Laine, J., Vasander, H., & Tuittila, E. S. (2007). Carbon gas exchange of a re-vegetated cut-away peatland five decades after abandonment. Boreal Environmental Research, 12, 177–190.
- Renou-Wilson F, Mueller C, Moser G, Wilson D (2016) To graze or not to graze? Four years greenhouse gas balances and vegetation composition from a drained and a rewetted organic soil under grassland. Agriculture Ecosystems & Environment 222:156–170
- Wang M, Wu J, Lafleur PM, Luan J, Chen H, Zhu X (2018) Can abandoned peatland pasture sequestrate more carbon dioxide from the atmosphere than an adjacent pristine bog in Newfoundland, Canada? Agricultural and Forest Meteorology 248:91–108
- D'Acunha B, Morillas L, Black TA, Christen A, Johnson MS (2019) Net ecosystem carbon balance of a peat bog undergoing restoration: integrating CO2 and CH4 fluxes from Eddy covariance and aquatic evasion with DOC drainage fluxes. Journal of Geophysical Research-Biogeosciences 124:884–901
- Beetz S, Liebersbach H, Glatzel S, Jurasinski G, Buczko U, Höper H (2013) Effects of land use intensity on the full greenhouse gas balance in an Atlantic peat bog. Biogeosciences 10:1067–1082
- Wilson, D., Farrell, C. A., Fallon, D., Moser, G., Müller, C., & Renou‐Wilson, F. (2016). Multiyear greenhouse gas balances at a rewetted temperate peatland. Global change biology, 22(12), 4080-4095.
- Schaller, C., Hofer, B., & Klemm, O. (2022). Greenhouse gas exchange of a NW German peatland, 18 years after rewetting. *Journal of Geophysical Research: Biogeosciences*, *127*(2), e2020JG005960.
- Evans, C., Morrison, R., Burden, A., Williamson, J., Baird, A., Brown, E., ... & Worrall, F. (2016). Final report on project SP1210: Lowland peatland systems in England and Wales–evaluating greenhouse gas fluxes and carbon balances.
- Evans, Chris ; Artz, Rebekka; Burden, Annette ; Clilverd, Hannah ; Freeman, Ben; Heinemeyer, Andreas; Lindsay, Richard; Morrison, Ross ; Potts, Jackie; Reed, Mark; Williamson, Jennifer . 2023 Aligning the Peatland Code with the UK Peatland Inventory [Final report]. London, Department for Environment, Food & Rural Affairs, 55pp.
- Juottonen, H., Hynninen, A., Nieminen, M., Tuomivirta, T., Tuittila, E. S., Nousiainen, H., ··· Fritze, H. (2012). Methane-cycling microbial communities and methane emission in natural and restored peatlands. Applied and Environmental Microbiology, 78, 6386–6389.
- Hendriks DMD, van Huissteden J, Dolman AJ, van der Molen MK (2007) The full greenhouse gas balance of an abandoned peat meadow. Biogeosciences 4:411–424
- Schrier-Uijl AP, Kroon PS, Hendriks DMD, Hensen A, Van Huissteden J, Berendse F, Veenendaal EM (2014) Agricultural peatlands: towards a greenhouse gas sink - a synthesis of a Dutch landscape study. Biogeosciences 11:4559–4576
- Jensen R, Herbst M, Friborg T (2017) Direct and indirect controls of the interannual variability in atmospheric CO2 exchange of three contrasting ecosystems in Denmark. Agricultural and Forest Meteorology 233:12–31
- Kandel TP, Lærke PE, Hoffmann CC, Elsgaard L (2019a) Complete annual CO2, CH4, and N2O balance of a temperate riparian wetland 12 years after rewetting. Ecological Engineering 127:527–535
- Poyda A, Reinsch T, Kluss C, Loges R, Taube F (2016) Greenhouse gas emissions from fen soils used for forage production in northern Germany. Biogeosciences 13:5221–5245
- Herbst M, Friborg T, Schelde K, Jensen R, Ringgaard R, Vasquez V, Thomsen AG, Soegaard H (2013) Climate and site management as driving factors for the atmospheric greenhouse gas exchange of a restored wetland. Biogeosciences 10:39–52
- Jacobs CMJ, Jacobs AFG, Bosveld FC, Hendriks DMD, Hensen A, Kroon PS, Moors EJ, Nol L, Schrier-Uijl A, Veenendaal EM (2007) Variability of annual CO2 exchange from Dutch grasslands. Biogeosciences 4:803–819
- Guenther A, Huth V, Jurasinski G, Glatzel S (2015) The effect of biomass harvesting on greenhouse gas emissions from a rewetted temperate fen. Global Change Biology Bioenergy 7:1092–1106
- Knox SH, Sturtevant C, Matthes JH, Koteen L, Verfaillie J, Baldocchi D (2015) Agricultural peatland restoration: effects of land-use change on greenhouse gas (CO2 and CH4) fluxes in the Sacramento-San Joaquin Delta. Glob Change Biol 21(2):750–765. https://doi.org/10.1111/gcb.12745
- Kalhori, A., Wille, C., Gottschalk, P., Li, Z., Hashemi, J., Kemper, K., & Sachs, T. (2024). Temporally dynamic carbon dioxide and methane emission factors for rewetted peatlands. Communications Earth & Environment, 5(1), 62.
- Tong, C. H. M., Peichl, M., Noumonvi, K. D., Nilsson, M. B., Laudon, H., & Järveoja, J. (2025). The Carbon Balance of a Rewetted Minerogenic Peatland Does Not Immediately Resemble That of Natural Mires in Boreal Sweden. Global change biology, 31(4), e70169.
- Peacock, M., Gauci, V., Baird, A. J., Burden, A., Chapman, P. J., Cumming, A., ... & Evans, C. D. (2019). The full carbon balance of a rewetted cropland fen and a conservation-managed fen. Agriculture, Ecosystems & Environment, 269, 1-12.
- Günther, A., Barthelmes, A., Huth, V., Joosten, H., Jurasinski, G., Koebsch, F., & Couwenberg, J. (2020). Prompt rewetting of drained peatlands reduces climate warming despite methane emissions. Nature communications, 11(1), 1644.
- Tiemeyer B, Freibauer A, Borraz EA, Augustin J, Bechtold M, Beetz S, Beyer C, Eblie M, Eickenscheidt T, Fiedler S, Förster C, Gensior A, Giebels M, Glatzel S, Heinichen J, Hoffmann M, Höper H, Jurasinski G, Laggner A, Leiber-Sauheitl K, Peichl-Brak M, Drösler M (2020) A new methodology for organic soils in national greenhouse gas inventories: Data synthesis, derivation and application. Ecol Indic 109:105838. https://doi.org/10.1016/j.ecolind.2019.105838

## Citations

Bedford, E. (2025). *Tesco PLC - statistics & facts*. https://www.statista.com/topics/3807/tesco-plc/#topicOverview

Carnell, E., & Tomlinson, S. (2025). *UK gridded population at 1 km resolution for 2021 based on Census 2021/2022 and Land Cover Map 2021*. NERC EDS Environmental Information Data Centre. https://doi.org/10.5285/7beefde9-c520-4ddf-897a-0167e8918595

DEFRA. (2025). Horticultural statistics 2024. In *Department for Environment, Food and Rural Affairs*. https://www.gov.uk/government/statistics/latest-horticulture-statistics

DNDC. (2018). *DeNitrification-DeComposition Model* (9.5). DNDC. https://dndc.sr.unh.edu

FAO. (2008). *Harmonized World Soil Database* (1.2). FAO. https://www.fao.org/soils-portal/data-hub/soil-maps-and-databases/harmonized-world-soil-database-v12/en/

FEPEX. (2022). *Exportación/importación españolas de frutas y hortalizas*. Ederación Española de Asociaciones de Productores Exportadores de Frutas, Hortalizas, Flores y Plantas. https://www.fepex.es/en-gb_datos-del-sector/exportacion-importacion-espa%C3%B1ola-frutas-hortalizas

Gargaro, M., Hastings, A., Murphy, R. J., & Harris, Z. M. (2024). A cradle-to-customer life cycle assessment case study of UK vertical farming. *Journal of Cleaner Production*, *470*, 143324. https://doi.org/10.1016/j.jclepro.2024.143324

Gargaro, M., Hastings, A., Murphy, R. J., & Harris, Z. M. (2025). A Comparative LCA of Field Grown Lettuce Versus Vertically Farmed Lettuce. *Food and Energy Security*, *14*(4). https://doi.org/10.1002/fes3.70117

Gobierno de Espana. (2021). *Analisis provincial de superficie, rendimiento y produccion 2021* (Vol. 1). https://www.mapa.gob.es/es/estadistica/temas/estadisticas-agrarias/agricultura/superficies-producciones-anuales-cultivos/

HortoInfo. (2024). *A pesar del Brexit, España sigue manteniendo el dominio del mercado de lechuga en Reino Unido con el 91’95% del total*. HortoInfo. https://hortoinfo.es/a-pesar-del-brexit-espana-sigue-manteniendo-el-dominio-del-mercado-de-lechuga-en-reino-unido-con-el-9195-del-total/

Li, C. S. (2000). Modeling trace gas emissions from agricultural ecosystems. *Nutrient Cycling in Agroecosystems*, *58*(1/3), 259–276. https://doi.org/10.1023/A:1009859006242

Met Office. (2024). *Met Office MIDAS Open: UK Land Surface Stations Data (1853-current)*. http://catalogue.ceda.ac.uk/uuid/dbd451271eb04662beade68da43546e1

Milà Canals, L., Muñoz, I., Hospido, A., Plassmann, K., McLaren, S., Edwards-Jones Project Manager, G., Hounsome, B., & Muñoz Almudena Hospido Katharina Plassmann Sarah McLaren, I. (2009). *LIFE CYCLE ASSESSMENT (LCA) OF DOMESTIC VS. IMPORTED VEGETABLES. Case studies on broccoli, salad crops and green beans Life Cycle Assessment (LCA) of Domestic vs. Imported Vegetables. Case studies on broccoli, salad crops and green beans Llorenç Milà i Canals*. http://www.surrey.ac.uk/CES

Natural England. (2024). *Peaty Soils Location*. https://naturalengland-defra.opendata.arcgis.com/datasets/Defra::peaty-soils-location-england/about

NX. (2025). *Tesco Distribution Centres*. The NX Group. https://thenxgroup.com/tesco-distribution-centres/

ONS. (2022). *The rise of the UK warehouse and the “golden logistics triangle.”*

ONS. (2024a). *Counties and Unitary Authorities (December 2022) Boundaries UK BUC*. Office for National Statistics. https://www.data.gov.uk/dataset/5bac6cf3-1969-4fe5-835b-f44b49d171f9/counties-and-unitary-authorities-december-2022-boundaries-uk-buc

ONS. (2024b). *Local Authority Districts (December 2021) Boundaries UK BGC*. Office for National Statistics.

ONS. (2025). *National population projections: 2022-based*. https://www.ons.gov.uk/peoplepopulationandcommunity/populationandmigration/populationprojections/bulletins/nationalpopulationprojections/2022based#:~:text=Back%20to%20table%20of%20contents,76.6%20million%20by%20mid%2D2047

Ordnance Survey. (2025). *OS Open Roads* (2025). Ordnance Survey. https://www.data.gov.uk/dataset/65bf62c8-eae0-4475-9c16-a2e81afcbdb0/os-open-roads1

SWC Maps. (2022). *Map of Supermarket Locations - UK Supermarket Map*. SWC Maps. https://maps.walkingclub.org.uk/shops/

Taft, H. E., Cross, P. A., Hastings, A., Yeluripati, J., & Jones, D. L. (2019). Estimating greenhouse gases emissions from horticultural peat soils using a DNDC modelling approach. *Journal of Environmental Management*, *233*, 681–694. https://doi.org/10.1016/j.jenvman.2018.11.113

Zhuang, Y., Lu, N., Shimamura, S., Maruyama, A., Kikuchi, M., & Takagaki, M. (2022). Economies of scale in constructing plant factories with artificial lighting and the economic viability of crop production. *Frontiers in Plant Science*, *13*. https://doi.org/10.3389/fpls.2022.992194

## 
